# Native *Regiella* Endosymbionts Provide Strong Parasitoid Protection With Limited Impacts on Fitness and Virus Transmission in *Myzus persicae*

**DOI:** 10.1111/1462-2920.70357

**Published:** 2026-06-21

**Authors:** Qiong Yang, Perran A. Ross, Alex Gill, Xinyue Gu, Neha Durugkar, Jia Chang, Owen J. Holland, Paul A. Umina, Christoph Vorburger, Torsten N. Kristensen, Ary A. Hoffmann

**Affiliations:** ^1^ Pest and Environmental Adaptation Research Group, School of BioSciences, the University of Melbourne Parkville Victoria Australia; ^2^ Section for Functional Ecology and Genomics, Department of Chemistry and Bioscience Aalborg University Aalborg Denmark; ^3^ Cesar Australia Brunswick Victoria Australia; ^4^ Department of Aquatic Ecology Eawag Dübendorf Switzerland

**Keywords:** *endosymbiont*, *Myzus persicae*, parasitoid protection, *Regiella*

## Abstract

A substantial literature has developed on facultative endosymbionts of insects, often portrayed as having large effects on their hosts, ranging from mutualistic to parasitic. The aphid endosymbiont *Regiella insecticola* occurs naturally in *Myzus persicae* and has potential biocontrol applications. Here, we examined the effects of native *Regiella* in two clones of *M. persicae* from Australia collected two decades apart, to assess whether these effects could explain the persistence of *Regiella* in natural populations. Genome sequencing revealed > 99.99% similarity between the *Regiella* strains from these clones. *Regiella* was stable in laboratory cultures and transmitted horizontally on excised leaves and intact plants. Fitness assays showed *Regiella* had modest costs to aphid reproduction but provided some benefits under heat stress, with no host‐plant‐specific effects. *Regiella* did not impact transmission of turnip yellows virus. *Regiella* provided strong protection against parasitism by two common parasitoid wasps, *Diaeretiella rapae* and *Aphidius colemani*. These findings indicate that while *Regiella* has limited fitness effects on *M. persicae* in the absence of parasitoids, its strong protection against parasitism underscores an important ecological function. However, these effects alone are not sufficient to explain the low incidence of *Regiella* in natural *M. persicae* populations, despite evidence for horizontal transfer.

## Graphical abstract

Native *Regiella insecticola* in *Myzus persicae* provides strong protection against parasitoid wasps but imposes only modest reproductive costs and does not affect virus transmission. Despite stable vertical and horizontal transmission, these effects alone do not explain the low natural prevalence of **Regiella** in field populations.

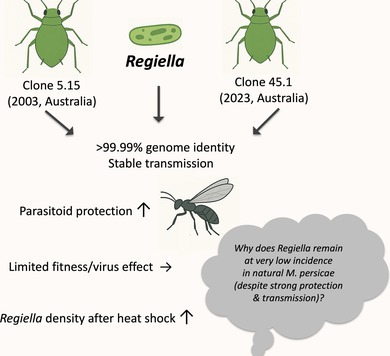

## 1 Introduction

Endosymbiotic bacteria are common in aphids and can spread rapidly through field populations via the proliferation of aphid clones carrying these endosymbionts (Smith et al. 2021), although this may partly reflect selection on aphid clones rather than directly on the endosymbionts (Zepeda‐Paulo et al. 2017; Leybourne 2025). Almost all aphids carry the obligate symbiont *Buchnera aphidicola* , which provides essential amino acids that aphids cannot obtain from the phloem of plants, but in addition may carry facultative endosymbionts whose distributions and frequencies are far more variable (Douglas 1998). However, endosymbiont distributions and frequency changes in natural populations are not yet well explained by their phenotypic effects measured under laboratory conditions. In particular, some endosymbionts reduce mummification (the formation of a hardened aphid body following successful development of a parasitoid larva) in the presence of parasitoids in the laboratory. As a result, the frequency of aphids carrying these endosymbionts may increase relatively quickly in cages (Oliver et al. 2008) and semi‐natural settings (Nell et al. 2024). However, such effects do not necessarily translate into a high frequency of the endosymbiont in natural populations even where parasitoids are present (Smith et al. 2021; Gimmi et al. 2023).

An understanding of the population dynamics of aphid endosymbionts requires multiple factors to be considered. These include potential fitness costs and benefits of the endosymbiont (Oliver et al. 2008; Hrcek et al. 2016) and its mode of transmission which may encompass both horizontal and vertical transmission (Kaech and Vorburger 2021; Gu et al. 2023). Various abiotic environmental effects may also influence endosymbiont density and frequency including temperature (Russell and Moran 2006), host plant species (Chang et al. 2025) and the host plants' water content (Díaz‐Hernández et al. 2025). Biotic components of the environment can extend beyond parasitoids and include endosymbiont effects on predation (Kovacs et al. 2017; Luo et al. 2022) and on viruses (Higashi et al. 2023), some of whose effects may in turn be dependent on the environment (Bitsch et al. 2025; Liu et al. 2019).

*Regiella insecticola* (hereafter referred to as *Regiella*) is a relatively common aphid endosymbiont (Sepúlveda et al. 2017; Zytynska and Weisser 2016) with potentially marked effects on aphid phenotypes. For instance, *Regiella* strains can influence life history traits including the incidence of winged forms (Zytynska and Weisser 2016), insect host and plant virus interactions (Higashi et al. 2023; Yu et al. 2025) and provide protection from fungal pathogens (Lukasik et al. 2013) and parasitism (Vorburger et al. 2010). Despite this, the dynamics of *Regiella* under field conditions are not well understood. In particular, the incidence of *Regiella* affecting parasitism was first described in the green peach aphid, *Myzus persicae* , from Victoria, Australia (Vorburger et al. 2010) where the endosymbiont is uncommon (Yang, Gill, et al. 2023). Fitness costs of *Regiella* seem low across aphid genotypes (Jamin 2019) and may not explain its low abundance. Other factors that may influence the incidence of *Regiella* remain to be examined, although it does not consistently influence predation by ladybirds (Bitsch et al. 2025). A possible explanation for rare but persistent occurrence could also be that the same symbiont is prevalent and ecologically important in another aphid species, from which it occasionally spills over into *M. persicae* . Although this hypothesis was not directly tested in the present study, our recent studies support this hypothesis: *Regiella* was not detected in *M. persicae* across a broad geographic range in Australia (Yang, Umina, et al. 2023), but it was found in several other aphid species in Victoria (Yang, Gill, et al. 2023). This raises the possibility that cross‐species spillover may contribute to the maintenance of *Regiella* at low frequencies in *M. persicae* .

Here we expand the characterisation of *Regiella* effects in *M. persicae* in several ways with the aim of obtaining comprehensive information relevant to its local dynamics, including how symbiont‐associated phenotypes respond to thermal stress conditions that aphid populations routinely experience across seasons. We collected data on the original strain of *Regiella* characterised by (Vorburger et al. 2010) as well as an independently sourced line of *M. persicae* carrying *Regiella* also collected from Victoria, Australia. We predicted that these *Regiella* strains would be genomically similar and that they occurred in the same clone. We also predicted that the fitness effects of *Regiella* in both lines would be similar and expanded fitness testing to thermally stressful conditions and across a range of host plant species. We tested for the first time horizontal transfer of the endosymbiont between aphids and also considered interactions between the endosymbiont and a plant virus (turnip yellows virus—TuYV) in the newly collected line in light of potential interactions between *Regiella* and plant viruses (Yu et al. 2025). TuYV is the most damaging virus to canola transmitted by *M. persicae* and also damages other brassica crops. Finally, we tested the prediction that *Regiella* in the new line would influence parasitism by *Diaeretiella rapae*, the main wasp parasite responsible for aphid parasitism in canola locally (Ward et al. 2021), as well as the wasp *Aphidius colemani*. Parasitism by both wasps has previously been shown to be reduced by *Regiella* in the original line of *M. persicae* (von Burg et al. 2008). The findings from these laboratory studies can help inform the field dynamics of this infection which may depend on interactions with seasonal conditions, parasitoids, host fitness effects, transmission and plant virus interactions.

## 2 Materials and Methods

### 2.1 Aphid Lines and Maintenance

Several *M. persicae* lines were used, including lines naturally infected with *Regiella* (5.15 (+) and 45.1 (+)), lines in which *Regiella* was removed by gentamicin microinjection (5.15 (−) and 45.1 (−)) and lines naturally free of facultative endosymbionts (8.1 (−) and 32.1 (−)) (Table 1). Aphid lines were distinguished using microsatellite markers, and endosymbiont status was confirmed by qPCR (‘Endosymbiont detection and quantification’). Lines were screened routinely, and the absence of other facultative endosymbionts was verified by 16S metabarcoding.

**TABLE 1:** *Myzus persicae* lines and the experiments in which they were used.

Aphid line	Facultative endosymbiont status	Experiments	Reference for origin
5.15 (+)	*Regiella insecticol*a (naturally occurring)	Fitness, endosymbiont density changes following thermal heat or cold shocks, heat knockdown, performance on different host plants, horizontal transfer, body colour.	Vorburger et al. (2010)
5.15 (−)	Absent (cured)	Control line for 5.15 (+)	Vorburger et al. (2010)
45.1 (+)	*Regiella insecticola* (naturally occurring)	Fitness, endosymbiont density changes following thermal heat shock, heat knockdown, performance on different host plants, horizontal tranfer, body colour, parasitism, TuYV transmission.	This study
45.1 (−)	Absent (cured)	Control line for 5.15 (−)	This study
8.1 (−)	Absent (natural)	*Regiella*‐free line for comparison to 45.1 lines in TuYV transmission.	This study
32.1 (−)	Absent (natural)	*Regiella*‐free line for comparison to 45.1 lines in TuYV transmission.	This study

The 5.15 (+) and 5.15 (−) lines were provided by Christoph Vorburger (Vorburger et al. 2010) and maintained at Aalborg University under controlled conditions (11°C–12°C, 16:8 h light: dark cycle) on excised radish ( *Raphanus sativus* ) leaves on 1% agar, following established protocols (Chirgwin et al. 2023). Periodically amplified on whole radish plants and age‐matched on excised leaves prior to experiments (Ross et al. 2025).

The 45.1 (+) line was established from a single female collected in 2023 from chilli near Burnley, Victoria, Australia (GPS: −35.83, 145.02). The 45.1 (−) line was produced by removing *Regiella* via gentamicin microinjection (Vorburger et al. 2010). Two additional *Regiella*‐free lines were included. The line (8.1 (−)) was collected from canola at Horsham, Victoria, Australia (GPS: −36.72, 142.18). The other line (32.1 (−)) was collected from eggplant at Bowen, Queensland, Australia (GPS: −20.01, 148.188).

Lines 45.1 (+), 45.1 (−), 8.1 (−) and 32.1 (−) were maintained on bok choy ( *Brassica rapa* subsp. chinensis) seedlings, transferred to fresh plants approximately every 2 weeks. All lines were cultured at 19°C ± 1°C under a 16:8 h light:dark photoperiod, with population amplification, age matching and experimental controls conducted under identical conditions at the University of Melbourne.

### 2.2 Plant Material

Plant material used in the experiments and culturing is summarised in Table S1. Bok choy was grown as the primary source plant material in maintenance and experiments for lines 45.1 (+), 45.1 (−), 8.1 (−) and 32.1 (−) at the University of Melbourne in Australia. Radish ( *Raphanus sativus* ‘French Breakfast 3’) was grown as the primary source plant material in maintenance and experiments for lines 5.15 (+) and 5.15 (−) at Aalborg University in Denmark.

In Australia, seeds were sown 3 cm deep into black pots (70 × 70 × 160 mm) filled with potting mixture (Osmocote premium potting mix, Scotts, VIC, Australia). Pots were housed in insect‐proof bug dorms (90 × 45 × 45 cm, 160 μm mesh) and maintained in a CT cabinet at 20°C ± 1°C, with a 16:8 h light: dark photoperiod under LED growth lights. Bok choy plants were used for aphid culturing at the five‐true‐leaf stage. Canola (var. Bonito), grown under identical conditions, was used for virus and host plant preference experiments at the second‐true‐leaf stage. Sage ( *Salvia officinalis* ) was purchased from Bunnings (Australia) and leaves of the umbrella plant (*Heptapleurum arboricola*) were collected from an urban dwarf umbrella tree.

In Denmark, bok choy, radish, potato ( *Solanum tuberosum* var. Anouk) and Persian clover ( *Trifolium resupinatum* var. *majus* Boss) were grown under conditions described previously (Ross et al. 2025).

### 2.3 Genome Sequencing and Comparative Analysis of *Regiella*

To assess strain similarity in *Regiella* between lines 5.15 (+) and 45.1 (+), whole‐genome sequences of *Regiella* were generated and compared. Genomic DNA was sequenced on an Illumina HiSeq platform and processed following the pipeline described previously (Yang et al. 2026). Reads were aligned to the *Regiella insecticola* reference genome (NCBI RefSeq GCF_963861935.1) using Bowtie2 v2.4.5, filtered as described in (Yang, Gill, et al. 2023). Pairwise single‐nucleotide genotypes were compared to estimate the proportion of identical sites across callable genomic positions. De novo genome assemblies for *Regiella* from lines 45.1 (+) and 5.15 (+) were also generated from short‐read whole‐genome data following the same procedures described previously (Yang et al. 2026).

### 2.4 Identification of Aphid Clonal Types Using Microsatellites

Aphid genotyping was performed on the four original lines (untreated with antibiotics), 5.15 (+), 45.1 (+), 8.1 (−) and 32.1 (−), across 10 microsatellite loci: M35, M37, M40, M49, M55, M63, M86, myz2, myz9 and myz25 following the DNA extraction and genotyping methods outlined in (Umina et al. 2022).

### 2.5 Endosymbiont Detection and Quantification

qPCR assays were used to confirm the presence or absence of *Regiella* and *Buchnera* endosymbionts and to measure their densities relative to a host gene. According to methods described previously (Yu et al. 2025; Yang, Gill, et al. 2023; Chirgwin et al. 2023). Three primer sets were used to amplify markers specific to *M. persicae* (actin, *Regiella* and *Buchnera*). Differences in Cp (ΔCp) between target endosymbiont and actin were averaged across 2–3 consistent replicate runs. Relative *Regiella* and *Buchnera* densities were determined by the 2^−ΔCp^ method.

### 2.6 Fitness Measurements With and Without Thermal Shocks (5.15 Lines)

This experiment was designed to quantify the effects of native *Regiella* infection on key fitness‐related traits of *M. persicae* under standard conditions and following exposure to thermal stress. By comparing aphids across control, cold and heat shock treatments, we aimed to assess whether *Regiella* affects development time, fecundity, longevity, body colour, or thermal tolerance in host aphids. Age‐matched nymphs (< 24 h old) from the 5.15 (+) and (−) lines were transferred in groups of 10 onto 20 mm radish leaf discs in Petri dishes (35 × 10 mm) with 30 replicate dishes per line and left at 19°C for 48 h. Dishes were randomly assigned to one of three temperature treatments: a heat shock at 36°C for 4 h, a cold shock at −3.5°C for 4 h, or a control maintained at 19°C with no thermal shock. These treatments were chosen based on pilot trials to determine heat and cold shock conditions that approached the lethal threshold but did not result in mortality in the 5.15 line. Petri dishes were sealed with Parafilm before exposure to the thermal shocks in a water bath containing antifreeze. The temperature ramped linearly from 19°C to the target temperature (36°C or −3.5°C) over 1 h, held for 4 h and then returned to 19°C for 1 h.

Aphids from the shocked and control treatments were transferred individually to 20 mm radish leaf discs in Petri dishes (35 × 10 mm) with 30 replicate dishes per line and treatment. Aphids were scored daily for mortality and fecundity by counting and removing their offspring. Leaf discs were checked and replaced at least twice per week or if the leaf quality had visibly deteriorated. Apart from being scored for fitness, body length and body colour measurements were made following the methods described elsewhere (Ross et al. 2025) whereby apterous adults were photographed with a dissecting microscope camera. Some aphids from these treatments but not measured for fitness were also used to score for the interaction between *Regiella* and *Buchnera* density (see below).

### 2.7 Fitness Measurements (45.1 Lines)

For the 45.1 (+) and (−) lines, we performed two experiments that were run under minor differences in rearing temperature (19.3°C and 20.6°C) and were therefore treated separately. Fitness assays followed the same general protocol as those carried out with the 5.15 lines, except that bok choy leaf discs were used instead of radish leaf discs and the thermal component was not included. A total of 150 replicate dishes were established per aphid line using bok choy leaf discs. Seventy‐five replicates were placed on the top shelf (19.3°C) and 75 on the bottom shelf (20.6°C). Body length was not measured in the 45.1 (+) and 45.1 (−) lines but we did consider body colour. Although the effects of thermal shocks on fitness were not considered for these lines, we did expose some aphids to a 36°C heat shock as described for the 5.15 lines to investigate impacts on *Regiella* and *Buchnera* endosymbiont densities (see below).

### 2.8 Heat Knockdown Time

We measured the heat knockdown time of all four *Regiella* (+) and (−) lines using water bath assays at a constant temperature of 40.5°C as described previously (Ross et al. 2025). In comparisons between the 5.15 (+) and 5.15 (−) lines we used 7‐d old nymphs reared on radish with 24 replicates per line. For the 45.1 (+) and (−) lines, 7‐d old nymphs were reared on bok choy with 44 replicates per line.

### 2.9 Horizontal Transfer

Understanding horizontal transmission is essential for predicting the persistence and spread of *Regiella* in natural aphid populations. Horizontal transfer of *Regiella* in *M. persicae* was assessed using two experimental designs following previously described methods (Gu et al. 2023) with minor modifications: (i) mixed aphid groups feeding on excised leaf discs and (ii) high‐density aphid groups feeding on live plants in clip cages (Figure S1).

#### 2.9.1 Leaf Discs in Petri Dishes

Two‐day‐old *Regiella* (+) donor and *Regiella* (−) recipient nymphs were placed together at a 1:1 ratio on excised bok choy leaf discs in 35 × 10 mm Petri dishes containing 1% agar (Figure S1A–C). Thirty replicate dishes were established for the 5.15 lines and 70 for the 45.1 lines, each containing a single donor–recipient pair. For the 45.1 lines, an additional 10 replicates were established with higher aphid densities (10 or 20 aphids per dish). Donor and recipient aphids were maintained together for 7 days (leaf discs replaced on day 4) before being stored in absolute ethanol for *Regiella* screening. Dishes in which aphids died were excluded. In the absence of horizontal transfer, 50% of aphids would therefore be expected to test positive for *Regiella*, corresponding to the original donor aphids. If more than 50% aphids tested positive for *Regiella*, we interpreted this as suggestive evidence of horizontal transfer. In a subset of replicates, aphids were allowed to produce F1 offspring, which were reared to adult stage and screened to assess whether *Regiella* persisted across generations. Positive and negative controls were conducted using donor and recipient aphids from the same infected or uninfected lines.

##### 2.9.1.1 Clip Cages on Live Plants

Twenty 45.1 (+) and twenty 45.1 (−) 2‐d‐old nymphs were mixed in a clip cage attached to the second true leaf of a living bok choy plant under the same maintenance conditions (Figure S1D). This setup was repeated three times, with aphids held together for 72 h before being stored in absolute ethanol. The 60 aphids obtained from the three replicates were pooled for *Regiella* screening, thus no biological replicates were available for statistical analysis. In the absence of horizontal transfer, 50% of aphids would be expected to test positive for *Regiella*. Detection frequencies exceeding this expectation were interpreted as evidence of transfer to recipient aphids.

### 2.10 Effects of *Regiella* on Parasitism by Two Parasitoid Species

This experiment tested whether *Regiella* infection confers resistance to parasitoid wasps and whether this protective effect is consistent across parasitoid species. This allowed us to evaluate the potential fitness benefits of *Regiella* under enemy pressure. We compared mummification frequencies between 45.1 (+) and 45.1 (−) aphids exposed to two parasitoid species: *Diaeretiella rapae* and *Aphidius colemani*. As the effects of *Regiella* in the 5.15 lines have been characterised previously (von Burg et al. 2008), therefore, experiments focused on the 45.1 lines.

We conducted 40 replicates per parasitoid species for each of the 45.1 (+) and 45.1 (−) lines. Parasitism enclosures were established using 7‐d‐old bok choy plants. Plants were enclosed within cups fitted with a basal hole and tubing sealed with cotton wool, allowed to establish for a further 7 days, and then seeded with two age‐matched adult apterous aphids. Enclosures were sealed with a second cup secured with mesh and Parafilm and maintained at 20°C ± 1°C under a 16:8 h light: dark cycle. After 24 h, adult aphids were removed and stored in absolute ethanol for subsequent *Regiella* screening.

Adults produced 8–17 nymphs per enclosure, which were allowed to develop to 2nd‐3rd instars over 48 h. Replicate with fewer than seven nymphs were excluded. This yielded 29 replicates from 45.1 (+) and 33 replicates from 45.1 (−) aphids exposed to *D. rapae* and 21 and 23 replicates, respectively, exposed to *A. colemani* .

Parasitoid mummies (Biological Services, Loxton, Australia) were held in groups of 200 in mesh‐covered vials with honey applied to 50% of the mesh. Once > 50% of the mummies had emerged, wasps were left for a further 24 h to allow mating. Wasps were exposed to CO_2_ and sexed. Following recovery, a single female wasp was introduced into each enclosure by aspirating the wasp into the bottom cup. Wasps were removed after 48 h and stored in absolute ethanol.

Aphid number was recorded 48 h after wasp removal (post‐parasitoid population), and again after a further 48 h (pre‐mummification population). Mummification scoring commenced 48 h later (6 days after parasitoid removal) and continued until no additional mummies were observed for 48 h, or until all aphids had mummified. Parasitism rate was calculated by dividing the number of mummies by the actual nymphal population.

$$\text{Parasitism rate}=\frac{\text{Number of mummies}}{\text{Post}-\text{parasitoid aphid population}-\text{Aphid mortality}},$$

where aphid mortality is defined as the number of aphids that died within the pre‐mummification period.

### 2.11 TuYV Maintenance and Detection

Turnip Yellow Virus (TuYV) isolate 5509, commonly found in Australian canola fields (Congdon et al. 2023), was provided by the Western Australian Department of Primary Industries. Virus cultures were maintained on canola seedlings (var. Bonito) grown in a CT room set at 20°C ± 1°C and 16:8 h light:dark photoperiod. After 18 d, 10 TuYV‐positive aphids (the third instar to adult) were transferred to a clip cage containing a second true leaf of bok choy for 72 h, after which aphids were removed and the plants allowed to develop for a further 16 d. Infection was confirmed by qPCR using a leaf sample (30 × 10 mm), and infected plants were either used in experiments or re‐used for virus maintenance.

To confirm the TuYV infection during the maintenance, total RNA was extracted from individual aphids or canola leaf discs using a Monarch Total RNA Miniprep Kit (NEB, Ipswich, USA) and reverse‐transcribed into cDNA using a high‐capacity cDNA reverse transcription kit (Thermo Fisher, Waltham, USA), following established protocols (Chirgwin et al. 2023). qPCR assays were conducted using TuYV‐specific primers (TuYV_CPF3: GGAATACTCAAGGCCTACCA; TuYV_CPR3: GGCTTTGTAATCCCGAACTT) and canola *β*‐actin as a reference gene (BnACTIN_F: ACGAGCTACCTGACGGACAAG; BnACTIN_R: GAGCGACGGCTGGAAGAGTA) (Jiang et al. 2021). Relative virus densities were calculated using the 2^−ΔCp^ method.

For virus transmission assays, TuYV levels in canola plants were quantified using DAS‐ELISA (LOEWE, Germany). Whole plants were homogenised in extraction buffer (1:3 w/v) with ceramic beads using a FastPrep‐24 system, and absorbance was measured at 405 nm. Samples were considered positive when absorbance values exceeded three times those of negative controls.

### 2.12 Impact of *Regiella* on the Transmission of *TuYV*

This experiment examined whether *Regiella* and aphid clonal type influence the transmission of TuYV by *M. persicae* . Four aphid lines, 45.1 (+), 45.1 (−), 8.1 (−) and 32.1 (−) were used across two trials. Virus source plants were established by inoculating canola plants as described above.

All experiments were conducted in a CT room at 19°C ± 1°C with a 16:8 h light: Dark photoperiod. In trial 1, 2‐d‐old nymphs from the 45.1 (+), 8.1 (−) and 32.1 (−) lines were placed onto TuYV‐infected canola plants using clip cages (10 aphids per plant) positioned over the second true leaf. Each line was replicated four times. Aphids were allowed a 72 h acquisition access period, after which a single aphid was transferred to an uninfected canola plant for a 48 h inoculation access period before removal. Plants were then maintained for 14 d to allow virus replication and subsequently harvested for TuYV quantification by ELISA. A total of 40 recipient plants were established per aphid line, yielding virus density data from 39 plants for the 8.1 (−) and 32.1 (−) lines and from 38 plants for the 45.1 (+) line. Ten TuYV‐uninfected plants were included as negative controls.

In trial 2, the same protocol was applied using 2‐d‐old nymphs from the 45.1 (+) and 45.1 (−) lines, with 20 aphids per TuYV‐infected source plant. Sixty replicate recipient plants were established per line, with virus density quantified in 55 plants for the 45.1 (+) line and 58 plants for the 45.1 (−) line. Ten TuYV‐uninfected plants were included as negative controls.

### 2.13 Host Plant Performance

Aphids from the 5.15 (+) and 5.15 (−) lines were measured for development time, fecundity and longevity on three host plants (bok choy, clover and potato) grown as described previously (Ross et al. 2025). Newly born nymphs (< 24 h old) from each line were placed individually onto 20 mm leaf discs in Petri dishes (35 × 10 mm), with 30 replicates per line‐host plant combination. Aphids were maintained and scored for traits following the fitness assay described above.

Aphids from the 45.1 (+) and 45.1 (−) lines were assessed for body length, development time, fecundity and longevity on three additional host plants not included in the 5.15 assays. Sage and umbrella plant, on which aphids performed poorly in pilot experiments, were selected to test whether *Regiella* conferred benefits under suboptimal host conditions, with canola included as a control. Experimental setup, replication (30 per line–host combination) and phenotyping followed the same procedures as described above.

### 2.14 Interaction Between *Buchnera* and *Regiella*

For the 5.15 lines, aphids that were exposed to thermal shocks (as described for the fitness measurement experiments above) but not measured for fitness traits were reared in groups of 10 on leaf discs until 15 days of age (12 d post stress), then stored in absolute ethanol. Fifteen to 30 individuals per aphid line and thermal shock treatment were then measured for relative *Regiella* and *Buchnera* densities using qPCR assays (‘Endosymbiont detection and quantification’).

For the 45.1 lines, we also explored relative densities of the two endosymbionts in the same way with a similar number of replicates using aphids that had not been exposed to fitness assays. In addition to considering the control treatment, we considered aphids that had been exposed to a 36°C heat shock at 3 days of age as described above for the 5.15 lines. Some of the aphids were tested directly after the heat shock (0‐d post treatment) when nymphs were 3 days old whereas others were tested 12 d post stress.

### 2.15 Statistics

Fitness traits (body colour, development time, body length, fecundity and longevity) were analysed using linear or generalised linear models to test for effects of *Regiella* status (+/−), temperature treatment and their interaction. These models were also used to analyse *Buchnera* and *Regiella* densities in relation to treatment, life stage and endosymbiont status, where treatment effects strongly influenced means and variances; analyses were conducted separately by treatment. Generalised linear models with a negative binomial error structure were used to analyse fecundity following heat stress and aphid performance on poor host plants (sage and umbrella plant), due to over‐dispersed count data. *Regiella* effects on parasitism were analysed using Mann–Whitney tests and TuYV transmission frequencies were analysed using contingency tests.

## 3 Results

### 3.1 *Regiella* Genomes and Aphid Clonal Types

For the genome sequencing, after alignment, genotype calling and filtering of the *Regiella* genomes, a total of 754,542 genomic positions were called and retained. *Regiella* genomes were maintained with almost identical similarity (754,539 positions; 99.9996%) between the 2 *M. persicae* lines.

We assembled the two *Regiella* genomes from two distinct clones. Although both assemblies are incomplete, the contigs exhibit overall genome‐wide synteny with no evidence of rearrangements (Figure S2).

Microsatellite genotyping was conducted on all four aphid lines using 10 DNA microsatellite markers, revealing that each represents a distinct multi‐locus clonal type. Multiple allelic differences were observed among the lines (Table S2). Although lines 5.15 and 45.1 shared identical alleles at 5 out of 10 loci, the presence of unique alleles at *myz9* and *M49A*, among others, clearly indicates that they are genetically distinct and belong to different clonal types. In contrast, lines 45.1 and 8.1 shared only one partial allele match at *M63A*, with 9 out of 10 loci differing completely, suggesting high genetic divergence. Lines 45.1 and 32.1 differed at 4 loci, confirming that they are also genetically distinct, though more closely related than line 8.1.

### 3.2 Fitness Measurements With and Without Thermal Shocks (5.15 Lines)

**FIGURE 1:**
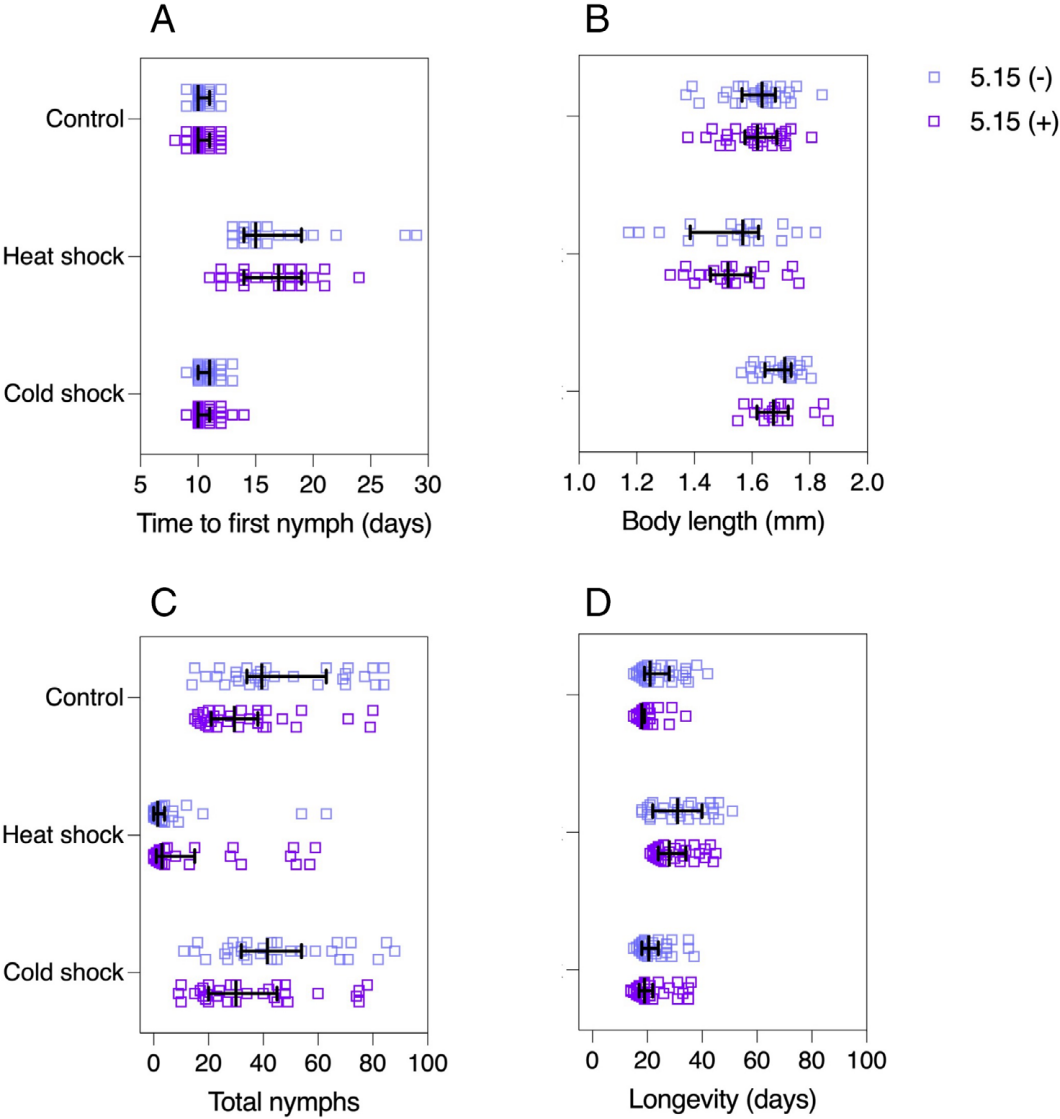
Fitness effects of *Regiella* in *M. persicae* lines 5.15 (+) and 5.15 (−) exposed to thermal shocks and maintained on excised radish leaf discs in Petri dishes. Aphids were reared at 19°C (control) or exposed to a heat (36°C) or cold (−3.5°C) shock for 4 h. Aphids were then reared individually and measured for (A) development time, (B) body length, (C) fecundity and (D) longevity. Dots represent data from individual aphids while vertical lines and error bars show medians and 95% confidence intervals respectively using a nonparametric, distribution‐free method based on order statistics.

We measured the fitness effects of *Regiella* status on the 5.15 lines with and without thermal shocks. The heat shock treatment extended development time and reduced fecundity substantially compared to the other treatments (Figure 1), and was therefore excluded from pairwise comparison for these traits. Development time was significantly affected by temperature treatment overall (generalised linear model, *F*_2,155_ = 119.36, *p* < 0.001, Figure 1A). Development time in the control and cold treatments overlapped but was slightly delayed in the cold treatment (*F*_1,115_ = 4.689, *p* = 0.032). Fecundity also differed significantly among temperature treatments overall (*F*_2,174_ = 40.69, *p* < 0.001, Figure 1C) but did not differ between the cold and control treatments (*F*_1,116_ = 0.003, *p* = 0.956). For body length, there was an overall treatment effect (*F*_2,126_ = 18.692, *p* < 0.001) due to a reduction in body length in the heat shock treatment (Figure 1B). Longevity was also affected by temperature treatment (*F*_2,170_ = 30.355, *p* < 0.001) due to an extended lifespan following heat shock (Figure 1D). Statistical analyses for this experiment and others involving multiple factors are provided in Table S3.

The *Regiella* status of the 5.15 lines had no effect on development time in the analysis of aphids from all treatments (*F*_1,155_ = 0.364, *p* = 0.547), the control and cold shock treatments (*F*_1,115_ = 0.253, *p* = 0.616) or from the heat shock treatment (*F*_1,40_ = 0.28, *p* = 0.600). The presence of *Regiella* influenced fecundity when the control and cold treatments were considered (*F*_1,115_ = 8.81, *p* = 0.004) due to a mean reduction in fecundity of 24% in 5.15 (+) aphids (Figure 1) but did not differ between 5.15 (+) and 5.15 (−) aphids after heat shock (generalised linear model, Wald *X* ^2^ = 2.291, df = 1, *p* = 0.130). Overall, longevity was decreased by the presence of *Regiella* (*F*_1,170_ = 4.78, *p* = 0.030) although the difference between means was relatively small (8.6%). *Regiella* did not influence body length (*F*_1,126_ = 0.026, *p* = 0.872). There were no significant two‐way interactions between *Regiella* status and treatment where these could be tested (Table S3), reflecting a consistent difference between the two lines under the control and cold shock treatments.

Aphid colour was not influenced by *Regiella* in terms of hue (*F*_1,126_ = 0.07, *p* = 0.792), lightness (*F*_1,126_ = 2.3677, *p* = 0.126) or saturation (*F*_1,126_ = 2.913, *p* = 0.090). However, the temperature treatments did influence lightness (*F*_1,126_ = 15.127, *p* < 0.001) and saturation (*F*_1,126_ = 137.436, *p* < 0.001) but not hue (*F*_1,126_ = 0.414, *p* = 0.662). There were no two‐way interactions among these terms (Table S3). Aphids from the heat shock treatment had a lower lightness score and a greater level of saturation (Figure S3).

### 3.3 Fitness Measures (45.1 Lines)

**FIGURE 2:**
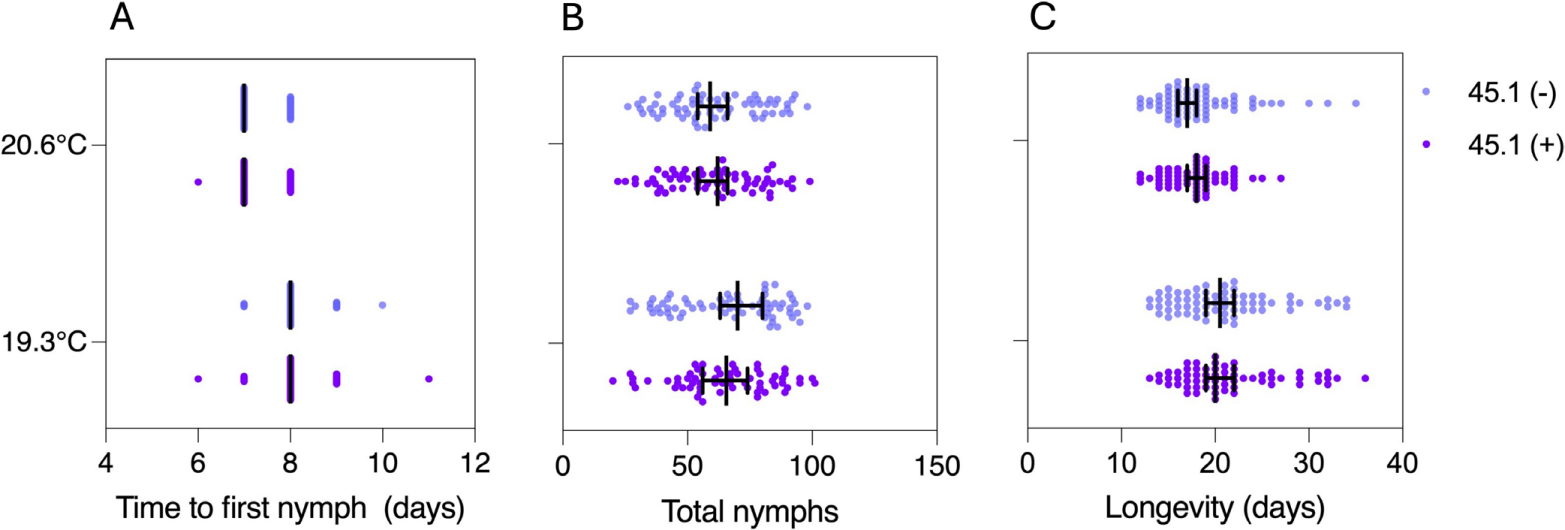
Fitness effects of *Regiella* in *M. persicae* lines 45.1 (+) and (−) maintained on excised bok choy leaf discs in Petri dishes. Repeat experiments were run at two temperatures (19.3°C and 20.6°C). Aphids were reared individually and measured for (A) development time, (B) fecundity and (C) longevity. Dots represent data from individual aphids while vertical lines and error bars show medians and 95% confidence intervals, respectively.

In the recently collected 45.1 line, we did not assess body length but did consider the other fitness traits (Figure 2) and body colour (Figure S4). There were effects of the minor differences in temperature on development time (*F*_1,255_ = 120.501, *p* < 0.001) and longevity (*F*_1,255_ = 28.449, *p* < 0.001) with the lower temperature extending these traits but having less impact on fecundity (*F*_1,255_ = 3.907, *p* = 0.049) (Figure 2). *Regiella* effects were not significant for development time (*F*_1,255_ = 0.033, *p* = 0.857), fecundity (*F*_1,255_ = 0.166, *p* = 0.684), or longevity (*F*_1,255_ = 0.001, *p* = 0.979) and there were no interaction effects (Table S3). Body colour was also unaffected by the endosymbiont (Figure S4).

### 3.4 Heat Knockdown Time

**FIGURE 3:**
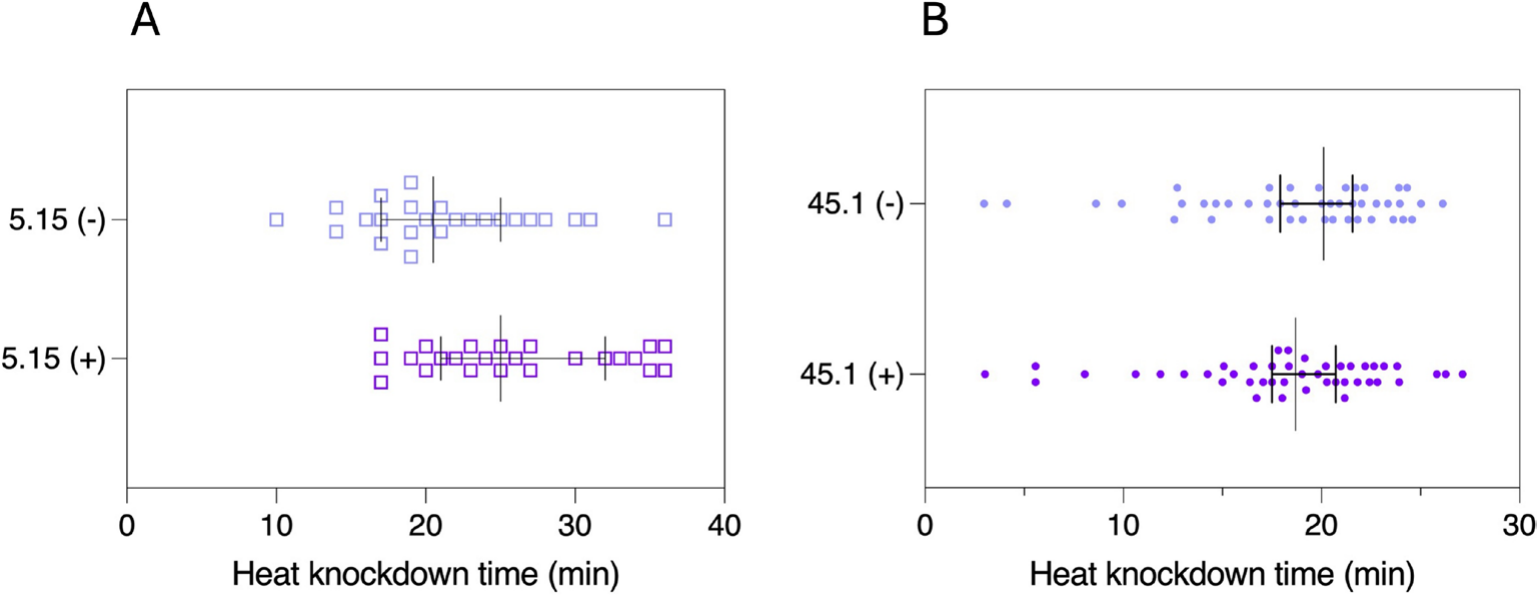
Heat knockdown time of *Regiella* (+) and (−) *M. persicae* lines from clones 5.15 and 45.1. Aphids were measured for their knockdown time in a water bath assay at a constant temperature of 40.5°C. Dots represent data from individual aphids while vertical lines and error bars show medians and 95% confidence intervals, respectively.

We used heat knockdown assays to estimate the impacts of *Regiella* on heat tolerance in the 5.15 (Figure 3A) and 45.1 (Figure 3B) lines. In the 5.15 lines, we found a significant effect overall of *Regiella* status (F_1,92_ = 5.591, *p* = 0.020), where 5.15 (+) aphids had a longer time to knockdown than 5.15 (−) aphids. These results point to a positive effect of *Regiell*a on heat tolerance in this clonal type. However, a comparison of the 45.1 lines (Figure 3B) showed no effect of *Regiella* status (*F*_1,86_ = 0.269, *p* = 0.606). Heat knockdown was therefore not influenced by *Regiella* in this line even though the assay was undertaken in the same way.

### 3.5 Horizontal Transfer

To test the potential for *Regiella* to spread to aphid lines without the endosymbiont, *Regiella* (+) donor aphids were paired with *Regiella* (−) recipient lines under varying group sizes and conditions. No *Regiella* was detected in negative controls (5.15 (−) or 45.1 (−) aphids only), while high densities (Cp < 25 and 2^−ΔCt^ > 1) were detected in all individuals in the positive controls (5.15 (+) or 45.1 (+) only), confirming the absence of background contamination and validating the detection method (Table 2).

**TABLE 2:** Horizontal transfer of *Regiella* between *Regiella* (+) donor and *Regiella* (−) recipient *M. persicae* individuals on bok choy leaf discs or live bok choy plants.

Donor	Recipient	Experimental design	Number of recipients (replicates)	Percent of recipients positive (±95% CIs)	Median *Regiella* Cp in recipients (±95% CIs)
5.15 (+)	5.15 (−)	Paired (leaf disc)	26	50	34.07 (32.27, 35.97)
5.15 (−)	5.15 (−)	Paired (leaf disc)	10	0	NA
5.15 (+)	5.15 (+)	Paired (leaf disc)	12	100	22.27 (19.91, 24.06)
45.1 (+)	45.1 (−)	Paired (leaf disc)	68	4.4	35.38 (34.18, 37.31)
45.1 (−)	45.1 (−)	Paired (leaf disc)	20	0	NA
45.1 (+)	45.1 (+)	Paired (leaf disc)	20	100	20.35 (19.69, 20.9)
45.1 (+)	45.1 (−)	Groups of 10 (leaf disc)	35 (7)	77.1 (49.1, 99.9)	32.71 (30.75, 34.54)
45.1 (+)	45.1 (−)	Groups of 20 (leaf disc)	60 (6)	91.7 (60.1, 94.0)	32.37 (32.5, 33.35)
45.1 (+)	45.1 (−)	Groups of 40 (whole plant)	60	56.7	33.25 (31.46, 34.29)

Abbreviation: NA, not applicable because no transfer possible.

In experimental treatments, at least 50% of aphids per replicate tested positive. Individuals with high‐density positive detections were inferred to be donors, while those testing positive at lower density (Cp > 25 and 2^−ΔCt^ < 0.5) were interpreted as recipients that had acquired *Regiella* via horizontal transfer. When single donor and recipient aphids were paired on a leaf disc, horizontal transfer occurred in a notable proportion of replicates. In the 5.15 line, 50% of recipient aphids tested positive, while in the 45.1 line, a lower transfer frequency (4.4%) was observed under the same conditions.

Increasing the group size of 45.1 aphids on a leaf disc enhanced the chance of horizontal transfer. When 10, 20, or 40 aphids were grouped together per leaf disc or clip cage at a 1:1 ratio of donor to recipient, the proportion of positive recipients was recorded as 77.14%, 91.67% and 56.67%, respectively (Table 2). This consistent pattern across treatments supports the conclusion that *Regiella* can be horizontally transmitted under close‐contact conditions.

Despite the high frequency of horizontal transfer in these assays, vertical transmission from mothers that acquired *Regiella* horizontally appeared rare. Of 69 F1 offspring from recipient mothers of the 45.1 line that had acquired *Regiella* horizontally, only one was scored as positive (Cp = 32), suggesting that cross‐generational transmission of horizontally acquired *Regiella* is uncommon. Offspring of this individual were not retained, and therefore the long‐term stability of this horizontally acquired infection across subsequent generations could not be assessed.

### 3.6 Effects of *Regiella* on Parasitism by Two Parasitoid Species

**FIGURE 4:**
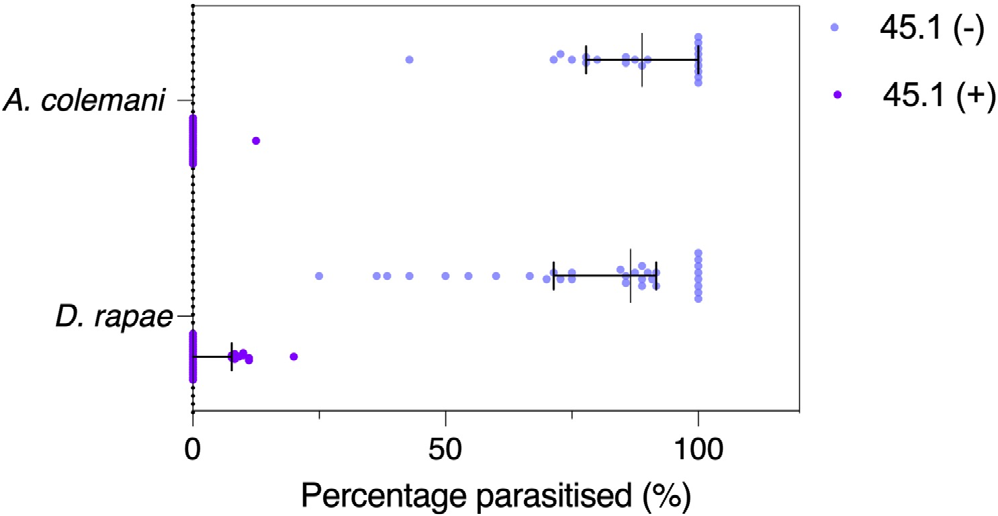
Percentage of 45.1 (+) and 45.1 (−) *M. persicae* parasitised by *D. rapae* and *A. colemani* . Each point represents a group of 8–17 aphids exposed to one parasitoid female; vertical lines and error bars show medians and 95% confidence intervals, respectively.

We tested the effects of *Regiella* status on parasitism as scored by mummification by *A. colemani* and *D. rapae* in the 45.1 (+) and (−) lines. There was strong protection from parasitism by the presence of *Regiella* regardless of parasitoid species tested (Figure 4). This result matches the results of Vorburger et al. (2010). Both comparisons of 45.1 (+) and (−) aphids were significant by non‐parametric Mann–Whitney tests (*p* < 0.001). For *Diaeretiella*, the median percentages were 0 (confidence intervals 0, 7.95) for *Regiella* (+) and 88.89 (95% confidence intervals 75.00, 91.67) for *Regiella* (−). For *Aphidius*, they were 0 (95% confidence intervals 0, 0) for *Regiella* (+) and 87.509 (95% confidence intervals 77.78, 100) for *Regiella* (−).

### 3.7 Impact of *Regiella* on Transmission of *TuYV*

**FIGURE 5:**
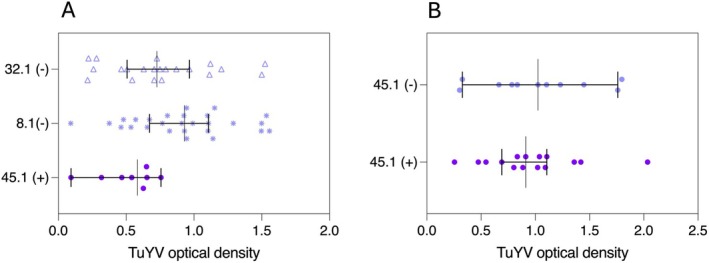
TuYV density in test canola plants when exposed to infected aphids via ELISA assay. In trial 1 (A), test plants were exposed to aphids from the 45.1 (+), 8.1 (−) and 32.1 (−) lines. In trial 2 (B), test plants were exposed to aphids from the 45.1 (+) and 45.1 (−) lines. Leaf material was collected from each test plant and screened for optical density. Dots represent data from individual positive test plants, while vertical lines and error bars show medians and 95% confidence intervals, respectively.

We compared the ability of different aphid lines to transmit TuYV to uninfected canola plants. The 45.1 (+) line had a similar TuYV transmission frequency between the two trials (18.4% and 27.3%). There were clonal differences in transmission frequency in trial 1 (*X* ^2^ = 18.62, df = 2, *p* < 0.001), due to a lower infection frequency in 45.1 (+) aphids (Table 3). There was also a significant effect of clonal type on TuYV density (*F*_2,53_ = 4.211, *p* = 0.020), with 45.1 (+) aphids having a lower optical density (Figure 5). However, there was no significant impact of *Regiella* on infection frequency (Table 3: *X* ^2^ = 1.649, df = 1, *p* = 0.199) or optical density (Figure 5: *F*_1,24_ = 0.107, *p* = 0.747) in trial 2 where the same clone was used.

**TABLE 3:** Infection frequency of TuYV in test canola plants when exposed to infected aphids from the 45.1 (+), 45.1 (−), 8.1 (−) and 32.1 (−) lines via ELISA assay.

Experiment	Aphid line	Number of plants tested	TuYV infection frequency in test plants (infected/total)
Trial 1	8.1 (−)	39	27/39
	32.1 (−)	39	21/39
	45.1 (+)	38	8/38
Trial 2	45.1 (+)	55	15/55
	45.1 (−)	58	10/58

### 3.8 Host Plant Performance

**FIGURE 6:**
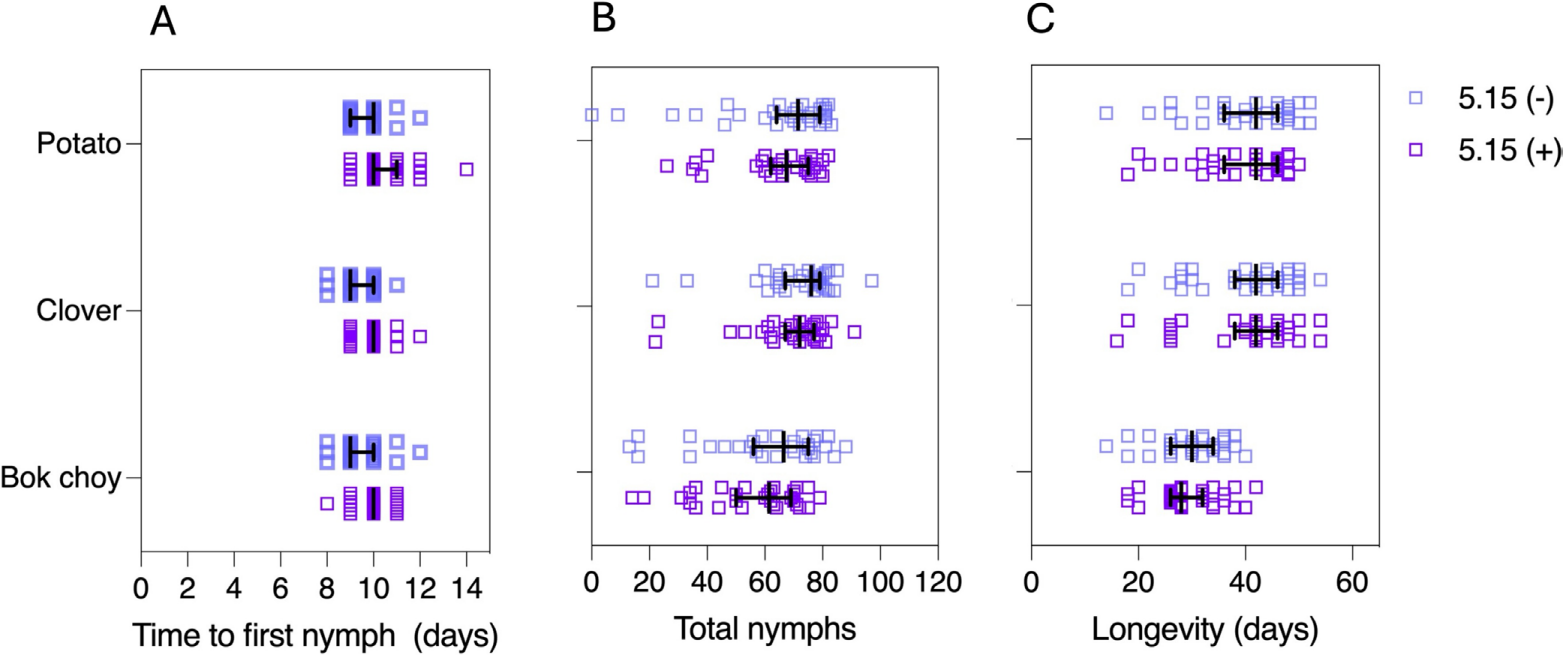
Fitness effects in *M. persicae* lines 5.15 (+) and 5.15 (−) reared on three host plants. Aphids were reared individually on excised leaf discs from potato, clover, or bok choy in Petri dishes. Aphids were then measured for (A) development time, (B) fecundity and (C) longevity. Dots represent data from individual aphids while vertical lines and error bars show medians and 95% confidence intervals, respectively.

To further investigate the effects of *Regiella* on *M. persicae* fitness, we performed additional experiments with clones 5.15 and 45.1 reared on different hosts. For clone 5.15, we found significant effects of host plant on development time (glm: *F*_2,173_ = 4.566, *p* = 0.012), fecundity (*F*_2,174_ = 6.701, *p* = 0.002) and longevity (*F*_2,174_ = 25.156, *p* < 0.001) where aphid performance on bok choy was poorer than on clover and potato (Figure 6). There was a significant effect of *Regiella* status on development time (*F*_1,173_ = 22.423, *p* < 0.001), with 5.15 (+) aphids taking 6.3% longer to develop. There were no such differences between 5.15 (−) and 5.15 (+) lines in fecundity and longevity (all *p* > 0.658). No two‐way interactions between 5.15 lines and host plant were significant, indicating that effects of *Regiella* were consistent across host plants for this clonal type.

**FIGURE 7:**
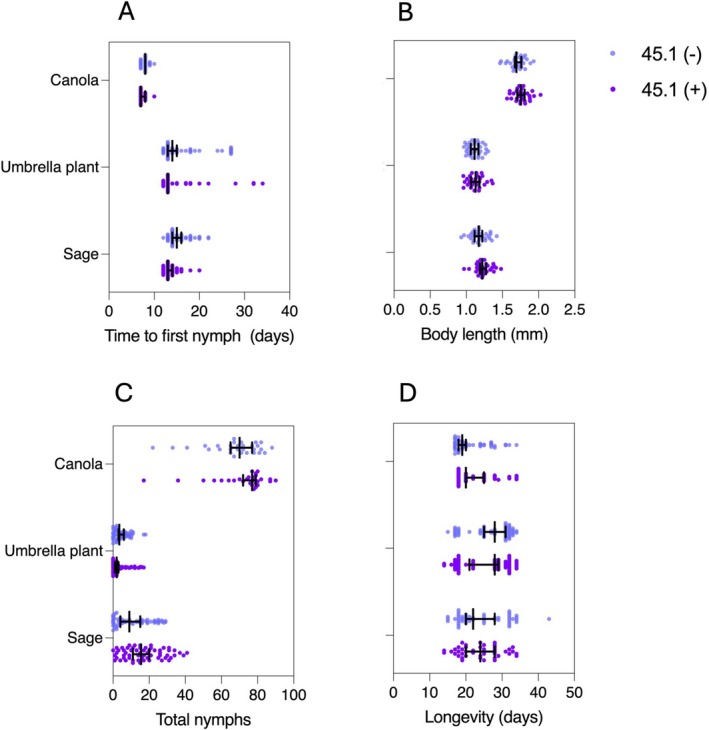
Fitness effects in *M. persicae* lines 45.1 (+) and 45.1 (−) reared on three host plants. Aphids were reared individually on excised leaf discs from canola, umbrella plant, or sage in Petri dishes. Aphids were then measured for (A) development time, (B) body length, (C) fecundity and (D) longevity. Dots represent data from individual aphids while vertical lines and error bars show medians and 95% confidence intervals, respectively.

For tests with clone 45.1, there were large differences in all traits between host plants (Figure 7), with the umbrella plant and sage resulting in longer development time, reduced body length, reduced fecundity and reduced longevity, confirming the low quality of these host plants compared to canola.

In contrast, effects of *Regiella* were relatively small or not detected. *Regiella* status affected body length (*F*_1,172_ = 6.13, *p* = 0.014), with 45.1 (+) aphids being 3.4% larger than 45.1 (−) aphids (Figure 7). However, there was no effect of *Regiella* status on longevity (*F*_1,243_ = 3.236, *p* = 0.073). For neither trait was there a two‐way interaction between *Regiella* status and host plant (Table S3).

For development time and fecundity, we analysed the effects of *Regiella* status separately through generalised linear models with a negative binomial distribution, as these traits had highly skewed distributions on poorer host plants. Development time was not significantly affected by *Regiella* status when aphids were reared on canola (Wald *X* ^2^ = 0.062, df = 1, *p* = 0.804), umbrella plant (Wald *X* ^2^ = 0.041, df = 1, *p* = 0.84) or sage (Wald *X* ^2^ = 0.38, df = 1, *p* = 0.537). *Regiella* also had no impact on fecundity when aphids were reared on canola (*F*_1,57_ = 1.28, *p* = 0.262), umbrella plant (Wald *X* ^2^ = 0.081, df = 1, *p* = 0.776) or sage (Wald *X* ^2^ = 2.039, df = 1, *p* = 0.153).

### 3.9 Interaction Between *Buchnera* and *Regiella*

**FIGURE 8:**
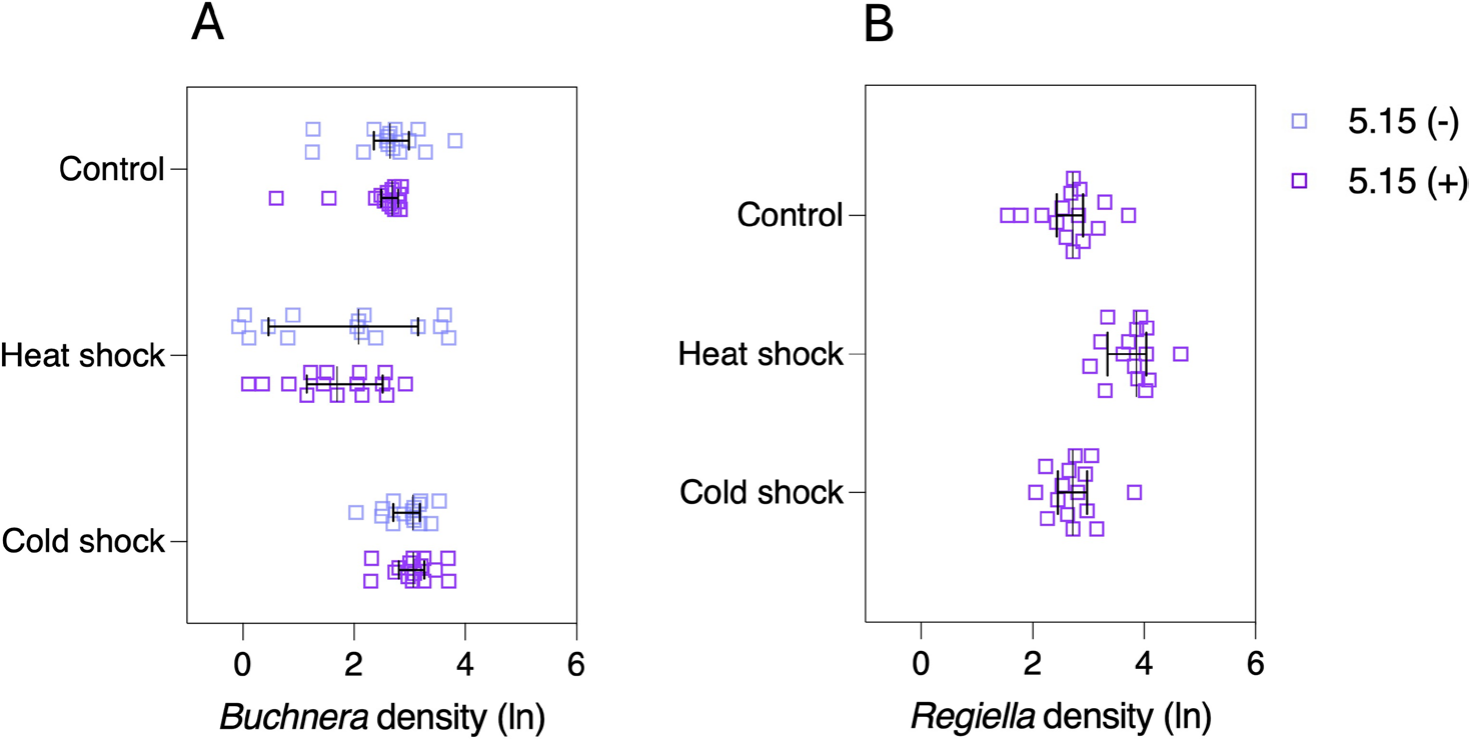
Endosymbiont densities of 5.15 (+) and (−) *M. persicae* exposed to thermal shocks. Aphids were reared at 19°C (control) or exposed to a heat (36°C) or cold (−3.5°C) shock. Aphids were then reared individually and measured for (A) *Buchnera* density and (B) *Regiella* density with qPCR assays 12 d after thermal shocks. Dots represent data from individual aphids while vertical lines and error bars show medians and 95% confidence intervals, respectively.

We measured endosymbiont densities in aphids from the 5.15 lines that were exposed to thermal shocks (see Figure 8). For *Buchnera* density, there was no significant effect of *Regiella* status (glm: *F*_1,84_ = 0.085, *p* = 0.891), indicating that the presence of this endosymbiont does not affect *Buchnera* abundance. We found a clear effect of temperature treatment (*F*_2,84_ = 19.729, *p* < 0.001) where aphids exposed to the heat shock treatment had lower *Buchnera* densities with a higher variance compared with the other treatments (Figure 8). This difference in variance between the heat shock treatment and control was significant by a Levene's test (*F*_1,58_ = 13.809, *p* < 0.001), whereas the difference between the cold shock and control treatment variances was not significant (*F*_1,58_ = 1.275, *p* = 0.264). There was no significant two‐way interaction between *Regiella* status and temperature treatment (Table S3). In the aphid lines that carried *Regiella*, we found significant effects of temperature treatment (*F*_2,42_ = 25.936, *p* < 0.001) on *Regiella* density. We observed that 5.15 (+) aphids experiencing a heat shock had increased *Regiella* densities compared to the other treatments but no shift in variance (Figure 8).

**FIGURE 9:**
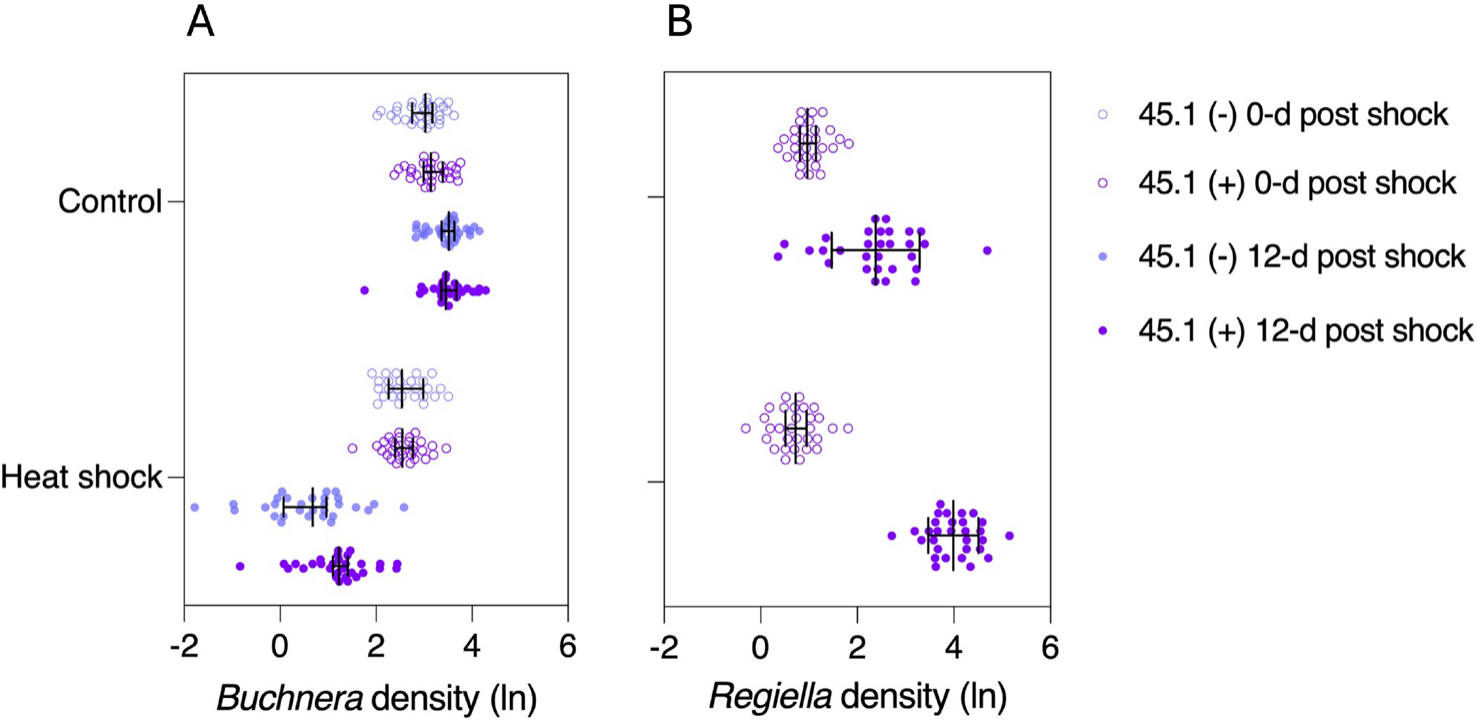
Endosymbiont densities of 45.1 (+) and (−) *M. persicae* exposed to a heat shock and tested at different life stages. Aphids were reared at 19°C (control) or exposed to a heat shock (36°C). Aphids were then measured for (A) *Buchnera* density and (B) *Regiella* density with qPCR assays either directly after the heat shock (Day 0) or reared individually and then measured 12 d later. Dots represent data from individual aphids while vertical lines and error bars show medians and 95% confidence intervals, respectively.

For the 45.1 lines, *Buchnera* densities were affected both by the aphid's life stage (*F*_1,232_ = 70.721, *p* < 0.001) and by the heat shock (*F*_1,232_ = 426.385, *p* < 0.001). In the control treatment, there was no interaction between the *Regiella* status and life stage (*F*_1,115_ = 0.773, *p* = 0.381) and no overall effect of the *Regiella* status (*F*_1,115_ = 2.636, *p* = 0.107) but there was an impact of life stage (*F*_1,115_ = 6.213, *p* < 0.001), with an increase in *Buchnera* density in older aphids (Figure 9). There was a significant interaction between life stage by *Regiella* status in the heat shock treatment (*F*_1,113_ = 3.206, *p* = 0.007), reflecting a reduction in *Buchnera* density at the 12‐d point of around 52% whereas day 0 densities remained similar. Main effects of life stage (*F*_1,113_ = 203.777, *p* < 0.001) and *Regiella* status (*F*_1,113_ = 5.843, *p* = 0.017) were also detected for *Buchnera* density under this treatment. A comparison of variances of the control and heat shock treatments showed a significant difference at the 12‐d point (*F*_1,58_ = 14.5542, *p* < 0.001) due to a much larger variance in the heat treatment, but no significant difference at day 0 (*F*_1,55_ = 0.402, *p* = 0.529) (Figure 9).

Consistent with the results from the 5.15 lines, *Regiella* density at the 12‐d point was higher in aphids exposed to the heat shock treatment compared with the control treatment (*F*_1,58_ = 70.906, *p* < 0.001). There was an overall interaction between treatment and life stage for *Regiella* density (*F*_1,115_ = 75.617, *p* < 0.001), reflecting a larger difference between the Day 0 and the 12‐d groups following heat shock (Figure 9). Life stage was significant in both the heat shock and control treatments (*p* < 0.001 in both cases). The variance between the heat shock and control treatments did not differ at Day 0 (*F*_1,55_ = 1.826, *p* = 0.182) or the 12‐d point (*F*_1,58_ = 3.343, *p* = 0.073), although there was a decrease in variance under the heat shock treatment observed (Figure 9).

## 4 Discussion

In this study, we characterised the effects of native *Regiella* in 2 *M. persicae* lines collected two decades apart for a range of key traits. Our genomic data confirm the prediction that the two strains are very similar, although the microsatellite data indicate that they come from different clones. The results show that effects of the two strains on host fitness are similar (Table 4) and may reflect a history of coevolution of this endosymbiont with its host. However, differences in heat shock tolerance across the strains need further testing, particularly as we found that heat shocks can suppress *Buchnera* but increase *Regiella* densities. Unlike the situation in *M. persicae* . *Regiella* reduces the effect of heat shock on life history traits and *Buchnera* density in the pea aphid ( *Acyrthosiphon pisum* ) (Heyworth et al. 2020; Clavé et al. 2022). And unlike in *M. persicae* , fitness costs of *Regiella* have been detected in other species, such as the corn aphid (*Rhopalosphum maidis*) (Liu et al. 2023).

**TABLE 4:** Summary of effects of *Regiella* detected in the 5.15 and 45.1 lines in this study.

Trait	Clone 5.15	Clone 45.1
Fitness traits*	No effect on development time or body length. Reduction in fecundity and longevity. No interaction with thermal shocks.	No effect on development time, fecundity, or longevity.
Body colour	No effect.	No effect.
Heat knockdown time	Increased tolerance.	No effect.
Horizontal transfer	50% on leaf discs with paired aphids.	4.4% on leaf discs with paired aphids. Higher rates of transfer when groups of aphids present in clip cages. Rare transfer to F1s.
Parasitism	(Protection)**	Strong protection from *A. colemani* and *D. rapae*.
Impact on TuYV transmission	Not tested.	No effect.
Host plant performance	Longer development time. No effect on fecundity or longevity.	No effect on development time, fecundity, or longevity. Small increase in body length.
Endosymbiont density	No effect on *Buchnera*. Increase in *Regiella* after heat shock.	No effect on *Buchnera* at 19°C. Higher *Buchnera* at 12 d after heat shock. Increase in *Regiella* after heat shock.

* For clone 5.15, it includes traits tested under control conditions and after thermal shocks.

** Finding from Vorburger et al. (2010), not retested here.

We acknowledge that aphids maintained on detached leaf discs may represent a sub‐optimal feeding environment relative to intact host plants. Fitness estimates should be interpreted as relative comparisons among treatments under standardised laboratory conditions rather than absolute measures of performance in the field. Nevertheless, leaf discs remain the easiest way of measuring individual aphid fitness at a high level of replication and can detect costs as well as benefits [e. g. 10]. They can be used alongside other measures such as clip cage confinement which has its own challenges (Martinez‐Chavez et al. 2024).

Our experiments on clone 45.1 support the prediction that *Regiella* has a protective effect against parasitism, based on previous work on clone 5.15 against both parasitoid species considered here (von Burg et al. 2008). Whether this translates into a large fitness advantage in the field remains unclear and will likely be seasonal with parasitoids common in the late season as temperature increases (Ward et al. 2021). *Regiella* may then also provide an advantage to the host under warm conditions as indicated for one of the strains tested for knockdown time. Given *Regiella* is uncommon in natural *M. persicae* populations in Australia (Yang, Gill, et al. 2023) and elsewhere (Beekman et al. 2022), despite being extremely common in aphids generally (Sepúlveda et al. 2017), there are clearly other factors influencing aphid fitness under field conditions. It may be that *Regiella* is currently associated with a very small number of clones that are less fit than other *M. persicae* clonal types. Within Australia, more than 300 multi‐locus clonal types have been characterised, yet > 90% of *M. persicae* populations collected from a wide range of agricultural and non‐agricultural fields across the country (Between 2010 and 2024) belong to one of three distinct clones (Umina et al. unpublished data). These have each evolved resistance to a large range of insecticides commonly used in Australia (Umina et al. 2022; Pym et al. 2022; Umina et al. 2014; Ward et al. 2024), enabling them to survive chemical sprays within cropping environments and, presumably, outcompete other clonal types that lack these resistance mechanisms. However, the horizontal transfer data collected here suggest the potential for inter‐clonal transfer of *Regiella* and we have found that *Regiella* from clone 45.1 transferred by microinjection to other *M. persicae* clones (and other aphid species) can lead to stable establishment of this endosymbiont (Gu et al. 2024).

Clearly there are costs of *Regiella* under field conditions that are not reflected in laboratory experiments. While *Regiella* can provide strong protection against *A. colemani* and *D. rapae*, this effect is not necessarily generalizable across a wider range of natural enemies that are typically present in the field, as shown for pea aphids (Hrcek et al. 2016), where in one case the fungal pathogens' protection provided by *Regiella* was reversed. In pea aphids exposed to predators, the fitness effects of *Regiella* can depend on the predator species (Kovacs et al. 2017), while in the cereal aphid ( *Sitobion avenae* ), parasitism effects from *Regiella* can also be species‐dependant (Luo et al. 2020). Nevertheless, for *Regiella* in *M. persicae*, protection was consistent across two parasitoid species, in line with earlier work (von Burg et al. 2008) and *Regiella* effects on predator susceptibility have also been found to extend to multiple species (Bitsch et al. 2025). Other factors, such as the presence of pathogenic fungi (Lukasik et al. 2013) and interactions with insect viruses (Higashi et al. 2023), could influence the fitness of aphids infected with *Regiella* under field conditions and these effects may also interact with climatic conditions (Bitsch et al. 2025; Liu et al. 2019), highlighting the difficulty of extrapolating from a limited number of experimental results.

The population dynamics of *Regiella* in *M. persicae* will also depend on horizontal transmission of *Regiella* across aphid populations. We have previously shown that horizontal transmission can have a substantial effect on the spread of another endosymbiont, *Rickettsiella viridis*, in *M. persicae* where initially low densities of this endosymbiont can increase over time (Gu et al. 2023). The current results suggest horizontal transfer of *Regiella*, particularly when aphid densities are high, though this is not the equivalent of transmission. Based on our 6 years of Australian endosymbiont survey data (Yang et al. 2023 and unpublished data), *Regiella* showed the broadest host distribution of an endosymbiont across 11 aphid species among the secondary endosymbionts surveyed. Nevertheless, it was only detected in a single line among 63 field‐collected *M. persicae* lines, namely line 45.1 used in this study. This suggests that any population effects associated with horizontal spread of *Regiella* in *M. persicae* may be weak. It may also depend on clonal type given that our paired leaf‐disc experiments suggested that line 5.15 showed substantially higher apparent horizontal transfer than line 45.1. The fact that the two *Regiella* genomes from the different *M. persicae* clones were nearly identical, despite being collected approximately 20 years apart, may indicate a relatively recent transmission event across clones.

It is still unclear whether *Regiella* can be transferred from aphid to aphid through plant tissue, as in the case of *Rickettsiella* (Gu et al. 2023), or if it requires direct contact between aphids (or their honeydew). It is possible that some observed cases of horizontal transfer correspond to aphids carrying *Regiella* DNA from transient contamination through honeydew or cornicle secretions from infected aphids. However, the detection of *Regiella* in an F1 offspring provides important proof‐of‐principle that horizontal transfer can, albeit infrequently, result in a vertically transmissible infection. This finding is difficult to explain by external contamination alone and suggests that establishment following horizontal acquisition is possible, although likely strongly constrained. Offspring from this individual were unfortunately not retained and therefore the long‐term stability of this horizontally acquired infection across subsequent generations could not be assessed. Further experiments could test transfer of native *Regiella* strains among different *M. persicae* clonal lineages and their subsequent persistence.

We found no evidence that *Regiella* influences the transmission of TuYV in *M. persicae* , despite earlier work showing the transmission of barley yellow dwarf virus by the bird‐cherry oat aphid ( *Rhopalosiphum padi* ) is impacted by the same *Regiella* strain present in clone 45.1 (Yu et al. 2025). In the same study, this *Regiella* strain leads to an increase in multiple fitness traits in *R. padi* (Yu et al. 2025), demonstrating endosymbiont transinfections can interact with hosts in novel ways, producing a different set of phenotypic effects than in their native host. This effect is well known for *Wolbachia* in mosquitoes where strains like *w*AlbB have little consistent impact on dengue transmission in their native *Aedes albopictus* environment but provide effective transmission blockage when transferred to the novel host *Aedes aegypti* (Ant et al. 2018). Although researchers have successfully undertaken interspecific transfers of endosymbionts like *Regiella* in aphids (Ward et al. 2024), this research could be extended to explore other desirable pest management traits, particularly those likely to be influenced by the immune system—given the interaction between endosymbiont density and immune gene expression (Goldstein et al. 2023).

In conclusion, we show that two closely related strains of *Regiella* isolated from Australian *M. persicae*, which are stable in laboratory cultures and capable of horizontal transfer, have limited impact on aphid fitness and body colour and inconsistent effects on heat knockdown tolerance. Across multiple host plant species, *Regiella* had only limited host plant‐specific effects and it did not impact transmission of TuYV in canola. We have separately investigated other factors like insecticide resistance (Dorai et al. 2024) and entomopathogen tolerance in *M. persicae* (Hoffmann et al. unpublished data) and found little advantage for aphids carrying *Regiella*. *Regiella* did provide strong and consistent protection against parasitoid wasps, although the impact of this in field situations remains unclear. Why native *Regiella* remains at a low incidence in natural *M. persicae* populations in Australia despite horizontal transfer remains an open question and will require more research within a field context.

## Author Contributions

**Qiong Yang:** conceptualization, methodology, data curation, investigation, validation, formal analysis, visualization, resources, writing – original draft, writing – review and editing, supervision, project administration. **Xinyue Gu:** methodology, data curation, investigation, validation, formal analysis, writing – review and editing. **Neha Durugkar:** methodology, writing – review and editing, investigation. **Perran A. Ross:** conceptualization, methodology, data curation, investigation, validation, formal analysis, supervision, visualization, project administration, resources, writing – original draft, writing – review and editing. **Alex Gill:** methodology, data curation, investigation, validation, project administration, resources, writing – review and editing, formal analysis. **Jia Chang:** methodology, software, data curation, investigation, formal analysis, writing – review and editing, writing – original draft. **Owen J. Holland:** methodology, software, data curation, investigation, formal analysis, writing – review and editing, writing – original draft. **Christoph Vorburger:** writing – review and editing, resources. **Paul A. Umina:** investigation, funding acquisition, writing – review and editing. **Ary A. Hoffmann:** conceptualization, methodology, investigation, validation, formal analysis, supervision, funding acquisition, project administration, resources, writing – original draft, writing – review and editing. **Torsten N. Kristensen:** funding acquisition, writing – review and editing, project administration, resources, investigation.

## Funding

This work was supported by Grains Research and Development Corporation (UOM1906‐002RTX, UOM2404‐006RT), Hort Innovation (ST23002).

## Ethics Statement

No specific ethical approval was required for this study because it involved invertebrates. All experiments were conducted in accordance with University guidelines.

## Conflicts of Interest

The authors declare no conflicts of interest.

## Supporting information


**Figure S1:** Experimental design for horizontal transmission experiments. (A–C) Set up using Petri dishes with leaf discs. A pair (A), a total of 10 (B) and a total of 20 (C) of *Regiella* (−) and *Regiella* (+) aphids were placed on bok choy leaf discs in Petri dishes at a 1:1 frequency. (D) Set up using clip cages on leaves of living bok choy plants.
**Figure S2:** Synteny plot of the two *Regiella* assemblies. Vertical bars represent contigs for the draft genome.
**Figure S3:** Body colour of apterous adult 5.15 (+) and (−) *M. persicae* exposed to thermal shocks and maintained on excised radish leaf discs in Petri dishes. Aphids were reared at 19°C (control) or exposed to a heat (36°C) or cold (−3.5°C) shock. Body colour was separated into three components: (A) hue, (B) saturation and (C) lightness. Dots represent data from individual aphids while vertical lines and error bars show medians and 95% confidence intervals, respectively.
**Figure S4:** Body colour of apterous adult 45.1 (+) and (−) *M. persicae* maintained on canola leaf discs in Petri dishes at 19°C. Body colour was separated into three components: (A) hue, (B) saturation and (C) lightness. Dots represent data from individual aphids while vertical lines and error bars show medians and 95% confidence intervals, respectively.
**Table S1:** Plant material and the experiments in which they were used.
**Table S2:** Clonal types of *M. persicae* lines based on allelic profiles of 10 DNA microsatellite markers. The allele sizes shown are inclusive of the universal primers used for fluorescent labelling following Blacket et al. (2012) and therefore may differ from other published studies.
**Table S3:** Statistical analyses for experiments with multiple factors analysed by general linear models. For each response variable, the model type, fixed factors, interaction terms, test statistics, degrees of freedom and *p* values are reported. Figure S2 (colour, 5.15 lines).

## Data Availability

Raw data from experiments associated with this work are archived through the University of Melbourne's FigShare server: 10.26188/31180492. *Regiella* genome sequence data, scripts and assemblies are also archived through the FigShare server: 10.26188/31198645.
