# Supplementary material for: Native Regiella Endosymbionts Provide Strong Parasitoid Protection With Limited Impacts on Fitness and Virus Transmission in Myzus persicae
Source: Environ Microbiol. 2026 Jun 21;28(6):e70357. doi: 10.1111/1462-2920.70357 (PMC13283851; doi:10.1111/1462-2920.70357)
Supplement: Supplementary file 1 — Figure S1: Experimental design for horizontal transmission experiments. (A–C) Set up using Petri dishes with leaf discs. A pair (A), a total of 10 (B) and a total of 20 (C) of Regiella (−) and Regiella (+) aphids were placed on bok choy leaf discs in Petri dishes at a 1:1 frequency. (D) Set up using clip cages on leaves of living bok choy plants. Figure S2: Synteny plot of the two Regiella assemblies. Vertical bars represent contigs for the draft genome. Figure S3: Body colour of apterous adult 5.15 (+) and (−) M. persicae exposed to thermal shocks and maintained on excised radish leaf discs in Petri dishes. Aphids were reared at 19°C (control) or exposed to a heat (36°C) or cold (−3.5°C) shock. Body colour was separated into three components: (A) hue, (B) saturation and (C) lightness. Dots represent data from individual aphids while vertical lines and error bars show medians and 95% confidence intervals, respectively. Figure S4: Body colour of apterous adult 45.1 (+) and (−) M. persicae maintained on canola leaf discs in Petri dishes at 19°C. Body colour was separated into three components: (A) hue, (B) saturation and (C) lightness. Dots represent data from individual aphids while vertical lines and error bars show medians and 95% confidence intervals, respectively. Table S1: Plant material and the experiments in which they were used. Table S2: Clonal types of M. persicae lines based on allelic profiles of 10 DNA microsatellite markers. The allele sizes shown are inclusive of the universal primers used for fluorescent labelling following Blacket et al. (2012) and therefore may differ from other published studies. Table S3: Statistical analyses for experiments with multiple factors analysed by general linear models. For each response variable, the model type, fixed factors, interaction terms, test statistics, degrees of freedom and p values are reported. Figure S2 (colour, 5.15 lines). [file EMI-28-e70357-s001.docx]

**Supplementary information**


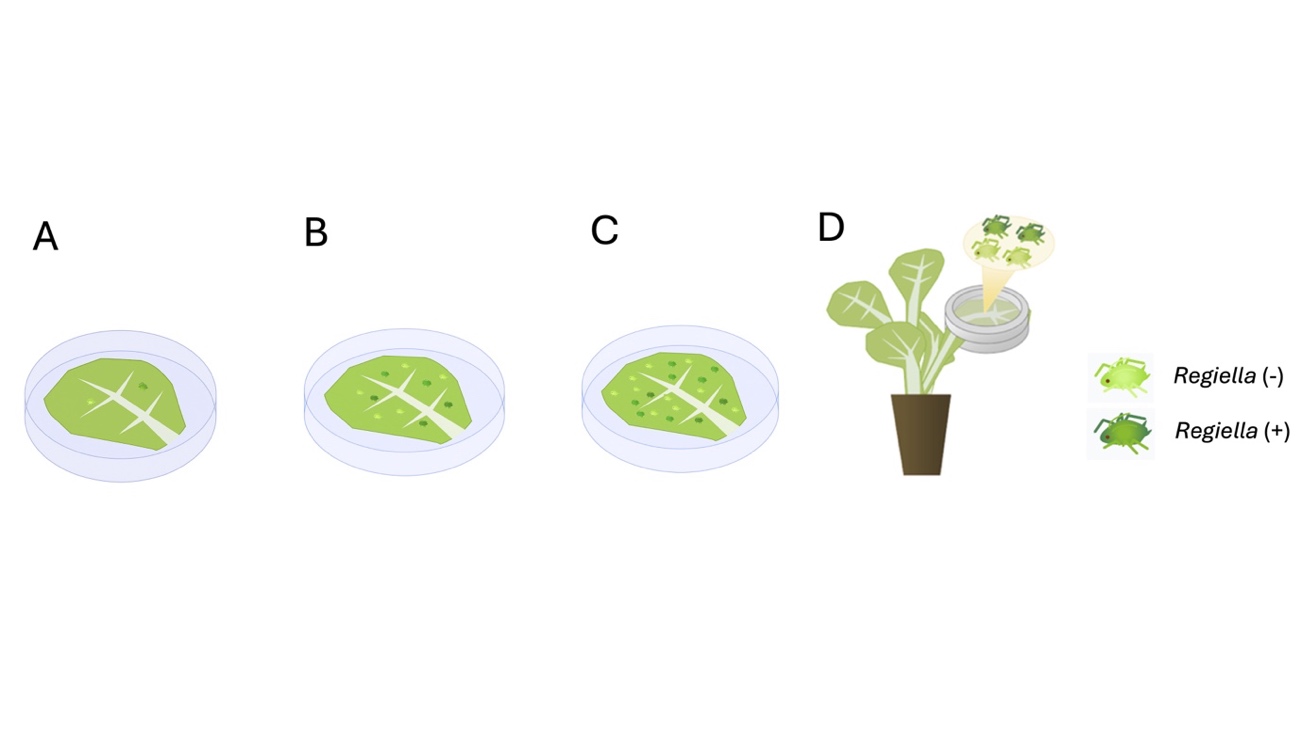


**Figure S1**. Experimental design for horizontal transmission experiments. A-C: Set up using Petri dishes with leaf discs. A pair (A), a total of ten (B) and a total of 20 (C) of *Regiella* (−) and *Regiella* (+) aphids were placed on bok choy leaf discs in Petri dishes at a 1:1 frequency. D: Set up using clip cages on leaves of living bok choy plants.


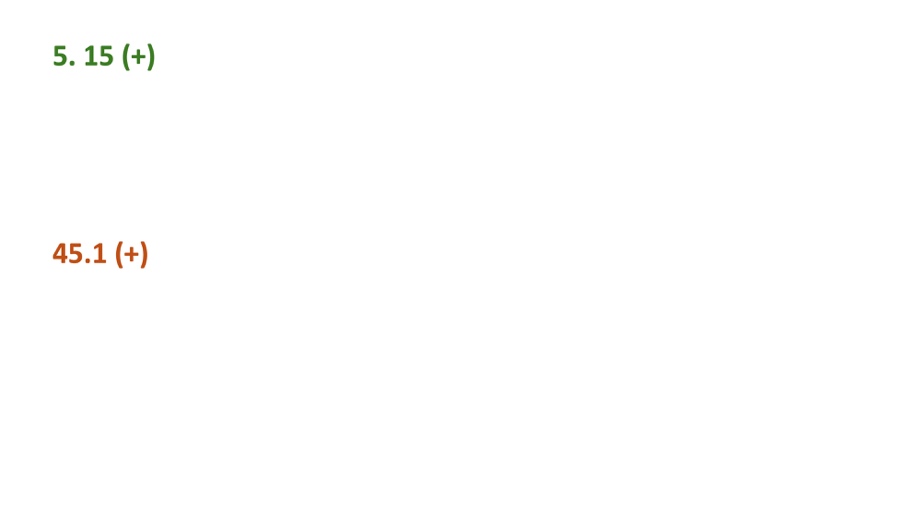

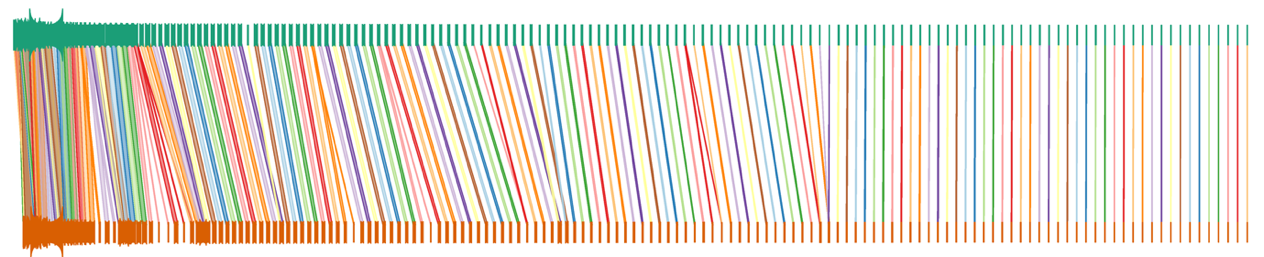


**Figure S2.** Synteny plot of the two *Regiella* assemblies. Vertical bars represent contigs for the draft genome.


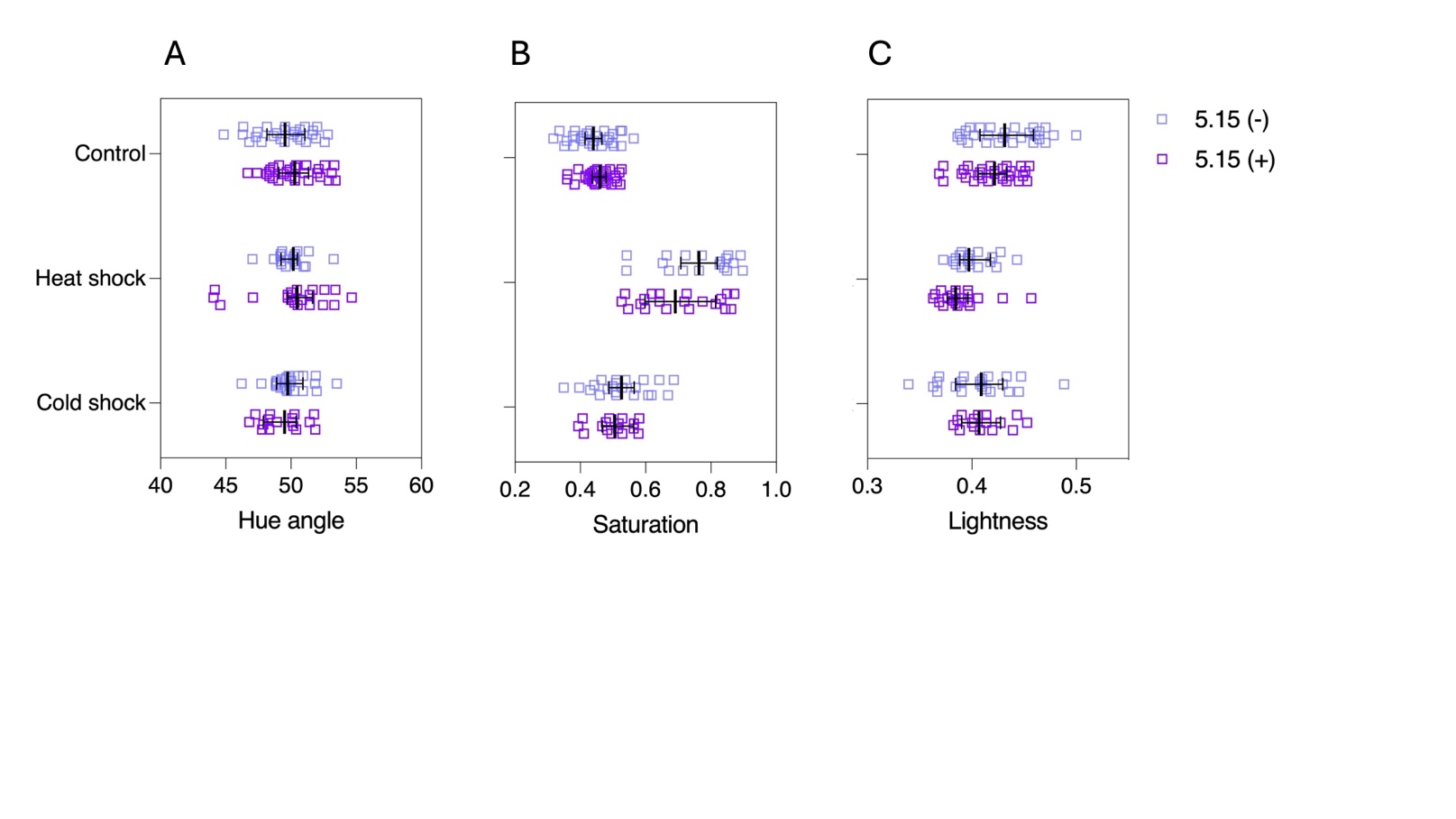


**Figure S3.** Body colour of apterous adult 5.15 (+) and (−) *M. persicae* exposed to thermal shocks and maintained on excised radish leaf discs in Petri dishes. Aphids were reared at 19°C (control) or exposed to a heat (36°C) or cold (-3.5°C) shock. Body colour was separated into three components: (A) hue, (B) saturation and (C) lightness. Dots represent data from individual aphids while vertical lines and error bars show medians and 95% confidence intervals, respectively.

**
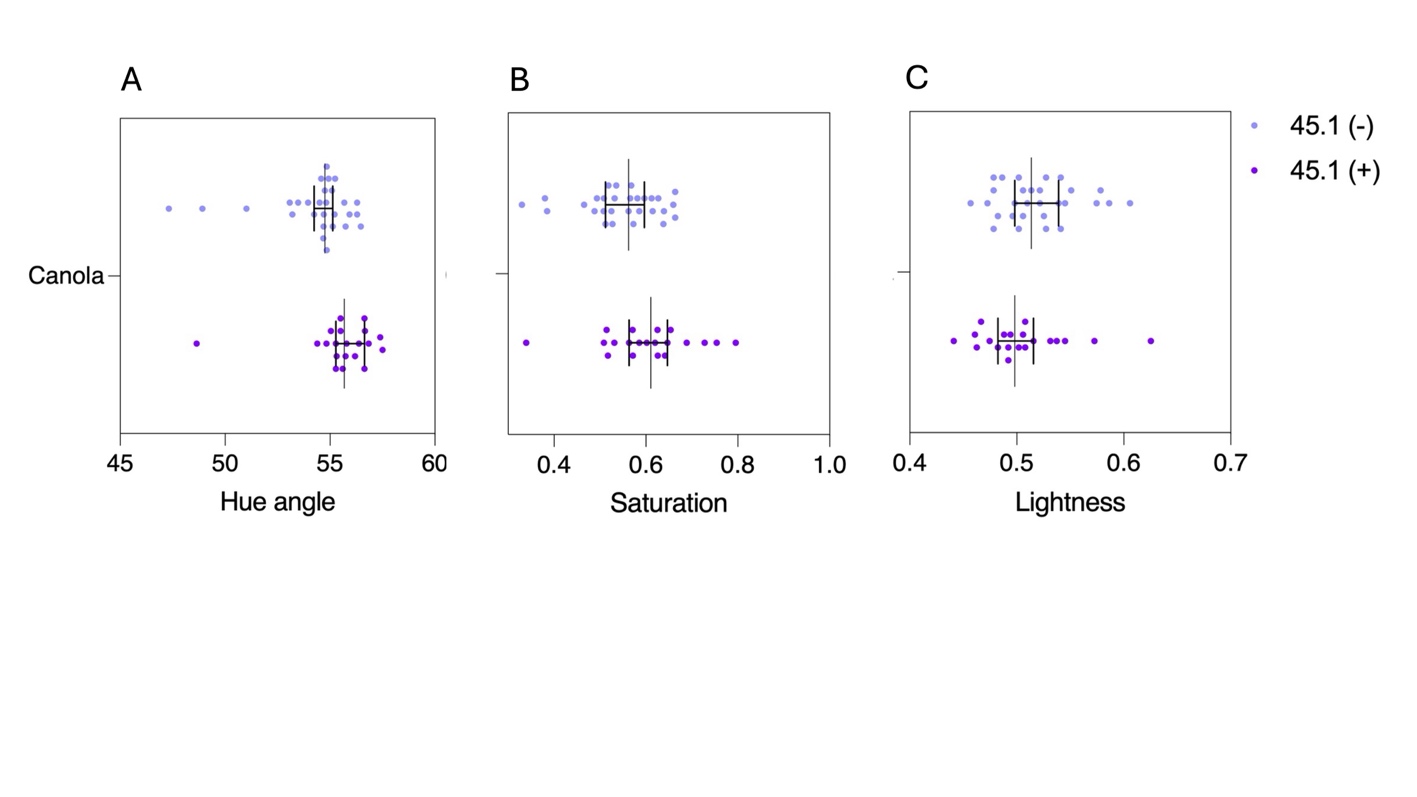
**

**Figure S4.** Body colour of apterous adult 45.1 (+) and (−) *M. persicae* maintained on canola leaf discs in Petri dishes at 19°C. Body colour was separated into three components: (A) hue, (B) saturation and (C) lightness. Dots represent data from individual aphids while vertical lines and error bars show medians and 95% confidence intervals, respectively.

**Table S1.** Plant material and the experiments in which they were used

| **Plant** | **Aphid line** | **Experiments and cultures** |
| --- | --- | --- |
| Bok choy | 5.15 (+), 5.15 (−) | Performance on different host plants, horizontal transmission. |
|  | 45.1 (+), 45.1(−), 8.1 (−), 32.1 (−) | Maintenance at University of Melbourne. |
|  | 45.1 (+), 45.1(−) | Fitness, heat knockdown, horizontal transmission, endosymbiont density changes following thermal heat shock. |
| Radish | 5.15 (+), 5.15 (−) | Maintenance at Aalborg University.  Fitness, endosymbiont density changes following thermal heat or cold shocks, heat knockdown, body colour. |
| Canola | 45.1 (+), 45.1(−) | TuYV maintenance at University of Melbourne.  Performance on different host plants, body colour. |
|  | 45.1 (+), 45.1(−), 8.1 (−), 32.1 (−) | TuYV transmission. |
| Potato | 5.15 (+), 5.15 (−) | Performance on different host plants. |
| Clover | 5.15 (+), 5.15 (−) | Performance on different host plants. |
| Umbrella plant | 45.1 (+), 45.1 (−) | Performance on different host plants. |
| Sage | 45.1 (+), 45.1 (−) | Performance on different host plants. |

**Table S2.** Clonal types of *M. persicae* lines based on allelic profiles of 10 DNA microsatellite markers. The allele sizes shown are inclusive of the universal primers used for fluorescent labelling following Blacket et al. (2012) and therefore may differ from other published studies.

| **Aphid line** | **Locus** | | | | | | | | | |
| --- | --- | --- | --- | --- | --- | --- | --- | --- | --- | --- |
|  | M40A | M63A | M86X | myz9 | M37A | M55A | myz2 | M35A | M49A | myz25 |
| 5.15 | 156/156 | 189/195 | 154/154 | 228/244 | 174/174 | 133/141 | 202/202 | 203/213 | 154/214 | 142/142 |
| 45.1 | 144/144 | 189/195 | 152/154 | 230/242 | 174/174 | 141/141 | 202/226 | 203/213 | 80/164 | 142/142 |
| 8.1 | 148/156 | 195/203 | 114/114 | 240/244 | 176/176 | 139/143 | 208/208 | 197/205 | 178/224 | 144/146 |
| 32.1 | 148/156 | 189/193 | 114/128 | 224/242 | 174/176 | 139/141 | 202/214 | 215/221 | 152/174 | 142/158 |

**Table S3.** Statistical analyses for experiments with multiple factors analysed by general linear models. For each response variable, the model type, fixed factors, interaction terms, test statistics, degrees of freedom and P values are reported.

| Relevant Figure, experiment | Response variable | Factors tested | MS, df | F, P value |
| --- | --- | --- | --- | --- |
| Figure 2 (fitness, 5.15 lines) | Development time | Temperature | 594.555, 2 | 119.363, <0.001 |
|  |  | Line | 1.812, 1 | 0.364, 0.547 |
|  |  | Interaction | 3.829. 2 | 0.384, 0.682 |
|  |  | Error | 4.981, 155 |  |
|  | Fecundity | Temperature | 1.20, 1 | 0.003, 0.956 |
|  |  | Line | 3808.13, 1 | 8.810, 0.004 |
|  |  | Interaction | 128.133, 1 | 0.296, 0.567 |
|  |  | Error | 432.24, 116 |  |
|  | Longevity | Temperature | 1572.606, 2 | 30.355, <0.001 |
|  |  | Line | 247.835, 1 | 4.781, 0.030 |
|  |  | Interaction | 71.282, 2 | 1.375, 0.256 |
|  |  | Error | 51.841, 170 |  |
|  | Body length | Temperature | 0.250, 2 | 18.692, <0.001 |
|  |  | Line | 0.000, 1 | 0.026, 0.872 |
|  |  | Interaction | 0.000, 2 | 0.006, 0.994 |
|  |  | Error | 0.013, 125 |  |
|  |  |  |  |  |
| Figure S2 (colour, 5.15 lines) | Colour (Hue) | Temperature | 1.662, 2 | 0.414, 0.662 |
|  |  | Line | 0.281, 1 | 0.070, 0.792 |
|  |  | Interaction | 4.836, 2 | 1.203, 0.304 |
|  |  | Error | 4.020. 126 |  |
|  | Colour (Lightness) | Temperature | 0.011, 2 | 15.127, <0.001 |
|  |  | Line | 0.002, 1 | 2.367, 0.126 |
|  |  | Interaction | 0.001, 2 | 1.785, 0.172 |
|  |  | Error | 0.001, 126 |  |
|  | Colour (Saturation) | Temperature | 0.940, 2 | 137.436, <0.001 |
|  |  | Line | 0.020, 1 | 2.913, 0.090 |
|  |  | Interaction | 0.019, 2 | 2.705, 0.071 |
|  |  | Error | 0.007, 126 |  |
| Figure 3 (fitness, 45.1 lines) | Development time | Temperature | 36.478, 1 | 120.501, <0.001 |
|  |  | Line | 0.010, 1 | 0.033, 0.857 |
|  |  | Interaction | 0.072, 1 | 0.238, 0.626 |
|  |  | Error | 0.303, 255 |  |
|  | Fecundity | Temperature | 1457.43, 1 | 3.907, 0.049 |
|  |  | Line | 61.82,1 | 0.166, 0.684 |
|  |  | Interaction | 13.25,1 | 0.036, 0.851 |
|  |  | Eror | 373.03, 255 |  |
|  |  |  |  |  |
|  | Longevity | Temperature | 632.19, 1 | 28.449, <0.001 |
|  |  | Line | 0.016, 1 | 0.001, 0.979 |
|  |  | Interaction | 17.129, 1 | 0.771, 0.381 |
|  |  | Error | 22.222, 255 |  |
| Figure 7 (host plant, 5.15 lines) | Development time | Plant | 3.318, 2 | 4.566, 0.012 |
|  |  | Line | 16.293, 1 | 22.423, <0.001 |
|  |  | Interaction | 0.373, 2 | 0.513, 0.600 |
|  |  | Error | 0.727, 173 |  |
|  | Fecundity | Plant | 2129.706, 2 | 6.701, 0.002 |
|  |  | Line | 355.606, 1 | 1.119, 0.292 |
|  |  | Interaction | 128.606, 2 | 0.405, 0.668 |
|  |  | Error | 31,820,174 |  |
|  | Longevity | Plant | 1920.867, 2 | 25.156, <0.001 |
|  |  | Line | 15.022, 1 | 0.197, 0.658 |
|  |  | Interaction | 0.956, 2 | 0.013, 0.988 |
|  |  | Error | 76.357, 174 |  |
| Figure 8 (host plant, 45.1 lines) | Longevity | Plant | 306.313, 2 | 8.433, <0.001 |
|  |  | Line | 117.531, 1 | 3.236, 0.073 |
|  |  | Interaction | 15.885, 2 | 0.437, 0.646 |
|  |  | Error | 36.324, 243 |  |
|  | Size | Plant | 6.299, 2 | 569.747, <0.001 |
|  |  | Line | 0.068, 1 | 6.13, 0.014 |
|  |  | Interaction | 0.009, 2 | 0.850, 0.429 |
|  |  | Error | 0.011, 172 |  |
| Figure 9 (5.15 lines, temperature) | *Buchnera* density | Temperature | 12.099, 2 | 19.729, <0.001 |
|  |  | Line | 0.052, 1 | 0.085, 0.772 |
|  |  | Interaction | 0.163, 1 | 0.266. 0.767 |
|  |  | Error | 0.613, 84 |  |
